# Dengue virus NS1 cytokine-independent vascular leak is dependent on endothelial glycocalyx components

**DOI:** 10.1371/journal.ppat.1006673

**Published:** 2017-11-09

**Authors:** Dustin R. Glasner, Kalani Ratnasiri, Henry Puerta-Guardo, Diego A. Espinosa, P. Robert Beatty, Eva Harris

**Affiliations:** Division of Infectious Diseases and Vaccinology, School of Public Health, University of California, Berkeley, CA, United States of America; Purdue University, UNITED STATES

## Abstract

Dengue virus (DENV) is the most prevalent, medically important mosquito-borne virus. Disease ranges from uncomplicated dengue to life-threatening disease, characterized by endothelial dysfunction and vascular leakage. Previously, we demonstrated that DENV nonstructural protein 1 (NS1) induces endothelial hyperpermeability in a systemic mouse model and human pulmonary endothelial cells, where NS1 disrupts the endothelial glycocalyx-like layer. NS1 also triggers release of inflammatory cytokines from PBMCs via TLR4. Here, we examined the relative contributions of inflammatory mediators and endothelial cell-intrinsic pathways. *In vivo*, we demonstrated that DENV NS1 but not the closely-related West Nile virus NS1 triggers localized vascular leak in the dorsal dermis of wild-type C57BL/6 mice. *In vitro*, we showed that human dermal endothelial cells exposed to DENV NS1 do not produce inflammatory cytokines (TNF-α, IL-6, IL-8) and that blocking these cytokines does not affect DENV NS1-induced endothelial hyperpermeability. Further, we demonstrated that DENV NS1 induces vascular leak in TLR4- or TNF-α receptor-deficient mice at similar levels to wild-type animals. Finally, we blocked DENV NS1-induced vascular leak *in vivo* using inhibitors targeting molecules involved in glycocalyx disruption. Taken together, these data indicate that DENV NS1-induced endothelial cell-intrinsic vascular leak is independent of inflammatory cytokines but dependent on endothelial glycocalyx components.

## Author summary

Dengue is the most prevalent mosquito-transmitted disease in humans and a significant public health issue worldwide. Severe dengue disease is characterized by vascular leak, which can lead to shock and potentially death. We previously demonstrated that nonstructural protein 1 (NS1), the only protein secreted from dengue virus (DENV)-infected cells, can both trigger vascular leak in mice when given systemically and increase permeability in human pulmonary endothelial cells via disruption of the endothelial glycocalyx-like layer, the molecular barrier that lines blood vessels. NS1 also triggers release of inflammatory cytokines from immune cells through activation of Toll-like receptor 4 (TLR4). Here, we explored the relative contributions of inflammatory molecules and the endothelial glycocalyx-like layer to NS1-mediated pathogenesis. Using cultured human dermal endothelial cells and mice genetically deficient for TLR4 or TNF-α receptor, we showed inflammatory signaling is not required for direct DENV NS1-mediated vascular leak. In contrast, inhibition of molecules involved in glycocalyx disruption blocked DENV NS1-induced vascular leak both in mice and *in vitro*. Altogether, our results indicate that disruption of endothelial glycocalyx components but not production of inflammatory cytokines is required for the direct action of DENV NS1 on endothelial cells and suggest potential molecular targets for treatment of severe dengue disease.

## Introduction

Dengue (DENV) is a mosquito-borne flavivirus that causes up to 390 million infections, 96 million cases of dengue, and ~500,000 hospitalizations annually. Infection with any of the 4 DENV serotypes (DENV1-4) results in a spectrum of disease from inapparent infection to classic dengue fever (DF) to dengue hemorrhagic fever/dengue shock syndrome (DHF/DSS), characterized by vascular leakage and shock. The DENV positive-strand 10.7 kb RNA genome encodes a polyprotein that is cleaved into 3 structural proteins and 7 non-structural proteins. DENV non-structural protein 1 (NS1) is synthesized by infected cells as a monomer (48 kDa), glycosylated in the ER, and released into the extracellular milieu as a hexamer (~310 kDa) [1–3]. Secreted DENV NS1 circulates in the blood during acute illness, and serum NS1 levels correlate with dengue disease severity, as does viral load (i.e., viremia) [4].

Under normal physiological conditions, the microvascular endothelium maintains a low permeability to fluids and molecules [5]. Disruption of the endothelial barrier can result in excessive leak across the endothelium, a phenomenon known as hyperpermeability. Clinically, this manifests as vascular leakage, where fluid accumulates in tissues after extravasating from the vasculature [5, 6]. Two of the primary determinants of endothelial barrier function are the endothelial glycocalyx and intercellular junctional complexes, such as tight and adherens junctions [7, 8]. The glycocalyx lines the luminal surface of the endothelium, protecting the underlying endothelial cells from shear forces and contributing to hemostasis, signaling, and blood cell-endothelial cell interactions [9]. Disruption of the glycocalyx has been shown to lead to vascular pathology and has been previously hypothesized to play a role in the pathogenesis of severe dengue disease [10], and modulation of the glycocalyx under inflammatory conditions is thought to contribute to various diseases [11].

DENV NS1 has been shown to play a role in viral replication [12, 13] and immune evasion [14, 15]. We recently showed that DENV NS1 can directly induce endothelial hyperpermeability *in vitro* and vascular leak *in vivo* in the absence of DENV infection, as well as lethally exacerbate an otherwise sublethal DENV infection [16]. We also demonstrated that DENV NS1 can disrupt the endothelial glycocalyx-like layer (EGL) *in vitro* through the activation of endothelial sialidases and the cathepsin L/heparanase pathway [17]. Further, glycocalyx components, such as heparan sulfate and chondroitin sulfate, have been shown to circulate at higher levels in the sera of DENV-infected patients than healthy controls [18, 19]. Others recently showed that NS1 can also act through Toll-like receptor 4 (TLR4) on mononuclear cells to induce secretion of vasoactive cytokines, and systemic inoculation of NS1 alone leads to significant increases in circulating levels of inflammatory cytokines in our mouse model [16].

In this study, we sought to evaluate the relative contributions of cytokine-driven inflammatory mechanisms and NS1-induced EGL degradation to NS1 pathogenesis of the endothelium. Using an *in vitro* model of endothelial permeability, we found that DENV NS1 triggers hyperpermeability independently of the pro-inflammatory cytokines TNF-α and IL-6. *In vivo*, we demonstrated that DENV NS1 induces localized vascular leak in the dermis of wild-type mice and that this effect is specific to DENV NS1 and independent of TLR4 and TNF-α signaling. Finally, we showed that DENV NS1-induced endothelial dysfunction is dependent on endothelial sialidases, cathepsin L, and heparanase both *in vitro* and *in vivo*. Taken together, our results indicate that DENV NS1 acts directly on the endothelium to induce vascular leak *in vivo* that is dependent on components of the glycocalyx.

## Results

### DENV2 NS1 triggers localized vascular leak in the dorsal dermis of mice

**Fig 1:**
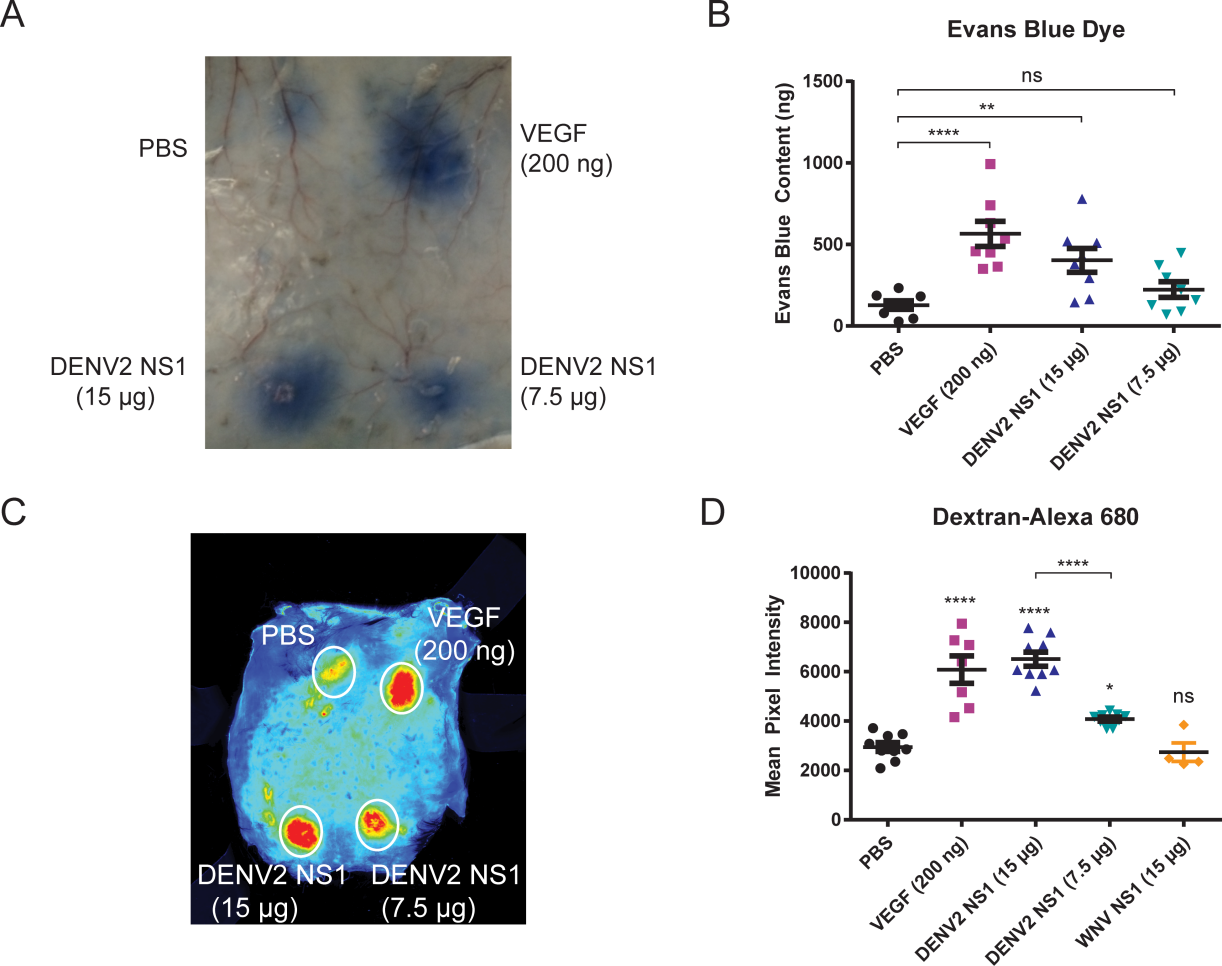
DENV2 NS1 triggers localized vascular leak in the dorsal dermis of mice. **(A-D)** Hair was removed from the dorsal dermis of mice, and mice were allowed to recover for 3 days. On the day of the assay, retro-orbital injections of **(A-B)** Evans Blue dye (EBD) or **(C-D)** Alexa Fluor 680-conjugated dextran were administered, followed by intradermal injections of PBS (black circles), 200 ng VEGF (purple squares), 15 μg DENV2 NS1 (blue triangles), 7.5 μg DENV2 NS1 (green triangles), and 15 μg WNV NS1 (orange diamonds). The dermis from each mouse was collected and processed two hours post-injection. **(A)** Representative image of mouse dorsal dermis following Evans Blue assay. **(B)** EBD was extracted from tissue in formamide for 24 hours at 56°C and quantified using a standard curve of EBD (30.5 to 500,000 ng/ml) using linear regression analysis. Data represent quantified EBD from 8 animals. **(C)** Representative image of mouse dorsal dermis following fluorescent dextran assay. **(D)** Dermises were scanned using a fluorescent detection system (LI-COR Odyssey CLx Imaging System) at a wavelength of 700 nm, and extravasated fluorescent dextran was quantified in tissue using Image Studio software (LI-COR Biosciences). Data represent the quantification of mean fluorescent intensity from mice in **(C)**: PBS (n = 9); VEGF (n = 7); DENV2 NS1–15 μg (n = 9); DENV2 NS1–7.5 μg (n = 9); WNV NS1 (n = 4). Data in **(B)** and **(D)** represent mean +/- SEM and were collected from 3 independent experiments. An ordinary one-way ANOVA with multiple comparisons to the PBS group using Dunnett’s multiple comparison test was used to determine significance of VEGF, DENV2 NS1, and WNV NS1. An unpaired, parametric, two-tailed t-test was used to determine significance between 15 μg of DENV2 NS1 and 7.5 μg of DENV2 NS1. ns = not significant, *P < 0.05, **P < 0.01, ****P < 0.0001.

*In vivo*, vascular permeability is often assessed by intravenous (IV) injection of small-molecule dyes, such as the albumin-binding Evans Blue dye (EBD); extravasation into tissues is then quantified by extracting dye in formamide and measuring absorbance at 620 nm. To measure the induction of vascular leak by DENV NS1 in the dermal endothelium, we removed hair from the dorsal side of wild-type C57BL/6 (B6) mice and administered a retro-orbital (RO) injection of EBD and four intradermal (ID) injections: phosphate-buffered saline (PBS) as a vehicle control, vascular endothelial growth factor (VEGF, 200 ng) as a positive control, and DENV2 NS1 (7.5 μg and 15 μg) **(Fig 1A).** Two hours post-injection, the dorsal dermis of the mice was removed, and equal areas of tissue were excised for EBD quantification. We found that VEGF and 15 μg of DENV2 NS1 induced vascular leak at levels significantly higher than PBS (p ≤ 0.0001, p = 0.0085, respectively) **(Fig 1C)**. These data demonstrate that DENV2 NS1 can induce vascular leak in the dermis of wild-type B6 mice.

To improve the sensitivity of the assay, we established a novel measure of vascular leak using IV injection of dextran molecules labeled with a fluorophore (Alexa Fluor 680), which can be quantified via fluorescent scanning. Similar to the traditional Evans Blue model, hair was removed from the dorsal side of wild-type B6 mice, fluorescent dextran was delivered RO, and the same four ID injections were administered as above **(Fig 1B)**. Using this model, we found that VEGF and both 7.5 μg and 15 μg of DENV2 NS1 induced vascular leak at levels significantly higher than PBS (*P* ≤ 0.0001, *P* = 0.0230, *P* ≤ 0.0001, respectively) **(Fig 1D)**. However, 15 μg of NS1 from West Nile Virus (WNV), a closely related flavivirus that causes encephalitis, did not trigger vascular leak in the dermis of wild-type B6 mice **(Fig 1D)**. NS1 from DENV1, 3, and 4 also induced vascular leak in our dermal model **(S1 Fig)**. Additionally, DENV2 NS1 was shown to induce vascular leak in the dermal endothelium of mouse ears using both Evans Blue and fluorescent dextran **(S2 Fig)**. Therefore, these data confirm our observations using Evans Blue dye and demonstrate a more sensitive method for detecting local vascular leak *in vivo*.

### Inflammatory cytokines TNF-α and IL-6 are not involved in DENV2 NS1-induced endothelial hyperpermeability *in vitro*

**Fig 2:**
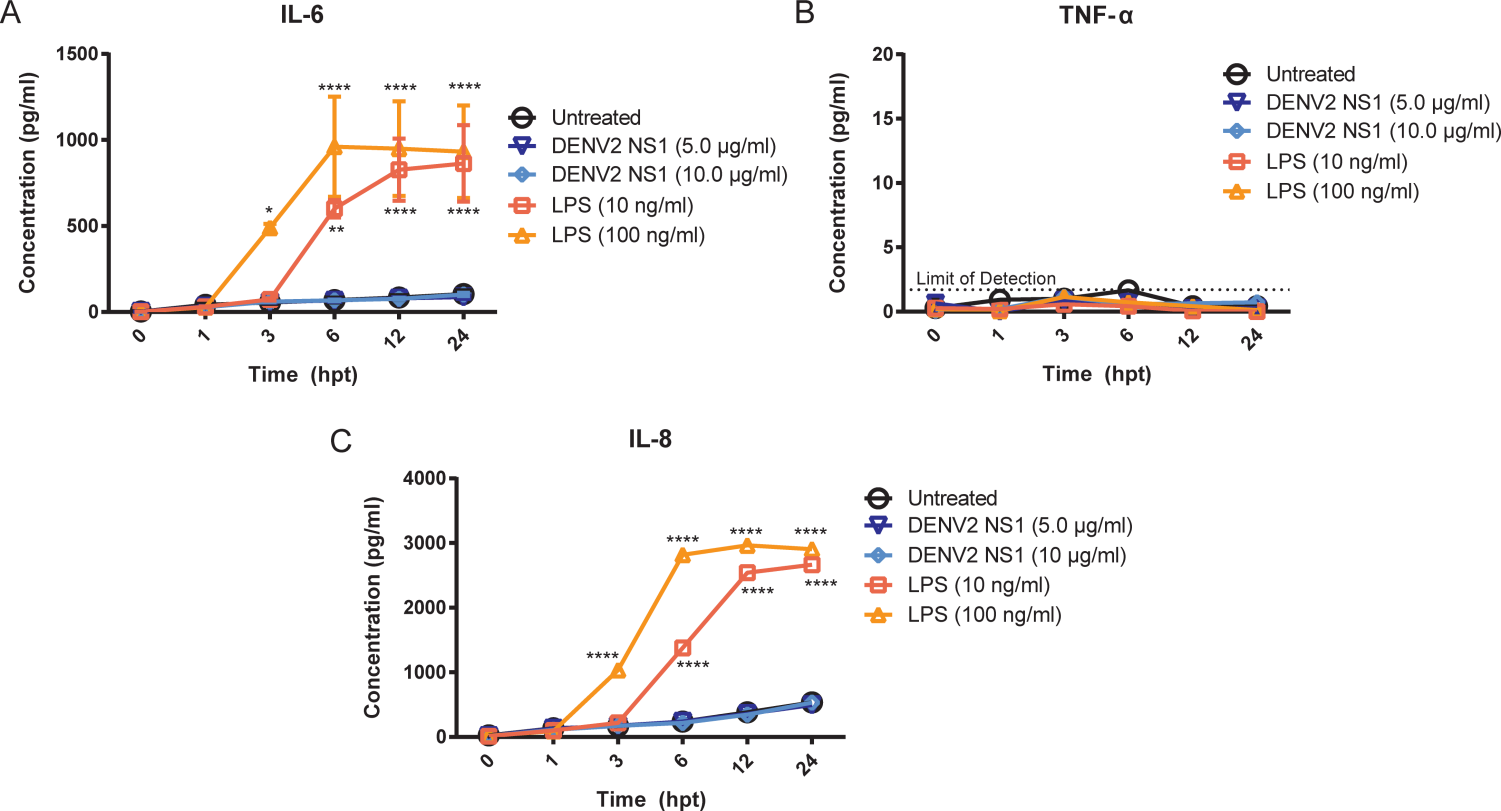
HMEC-1 do not produce the inflammatory cytokines IL-6, TNF-α, or IL-8 in response to DENV2 NS1 stimulation *in vitro*. **(A-C)** HMEC-1 were stimulated with LPS (10 or 100 ng/ml; red squares and orange triangles, respectively) or DENV2 NS1 (5 or 10 μg/ml; dark blue triangles and light blue diamonds, respectively), and supernatant was collected at 0, 1, 3, 6, 12, and 24 hours post-treatment. Untreated HMEC-1 monolayers were used as a control (black circles). ELISAs for **(A)** IL-6, **(B)** TNF-α, and **(C)** IL-8 were performed on all samples. All data shown represent the mean +/- SEM and were collected from two independent experiments. A repeated measure two-way ANOVA with multiple comparisons to the untreated group using Dunnett’s multiple comparison test was used to determine significance of treatment with LPS (10 and 100 ng/ml) or DENV2 NS1 (5 and 10 μg/ml). *P < 0.05, **P < 0.01, ****P < 0.0001.

In addition to the direct NS1-mediated hyperpermeability we showed in endothelial cells that is due in part to disruption of the EGL [17], Modhiran et al. reported that DENV NS1 can trigger release of vasoactive cytokines from peripheral blood mononuclear cells (PBMCs) via activation of TLR4 [20]. To determine whether inflammatory cytokines are involved in DENV2 NS1-mediated endothelial hyperpermeability *in vitro*, we first determined whether human dermal endothelial cells produce specific cytokines in response to DENV2 NS1. We stimulated the human dermal microvascular endothelial cell line HMEC-1 with 5 μg/ml and 10 μg/ml of DENV2 NS1 and collected supernatant at 0, 1, 3, 6, 12, and 24 hours post-treatment. Untreated HMEC-1 were used as a steady-state control, and HMEC-1 treated with 10 ng/ml or 100 ng/ml of lipopolysaccharide (LPS) were used as a positive control. We found that HMEC-1 did not produce IL-6 in response to DENV2 NS1 but did in response to both concentrations of LPS in a dose-dependent manner **(Fig 2A)**. HMEC-1 did not produce detectable levels of TNF-α in response to either DENV2 NS1 or LPS **(Fig 2B)**; LPS only stimulates production of TNF-α from endothelial cells in the presence of a secondary inflammatory signal [21]. Additionally, IL-8, a chemokine known to play a role in the inflammatory response, was only produced in response to LPS and not DENV2 NS1 in HMEC-1 (**Fig 2C)**. Similar results for IL-6, TNF-α, and IL-8 were obtained when human pulmonary microvascular endothelial cells (HPMEC) were studied **(S3 Fig)**. This suggests that endothelial cells do not produce inflammatory cytokines in response to DENV2 NS1 *in vitro*.

**Fig 3:**
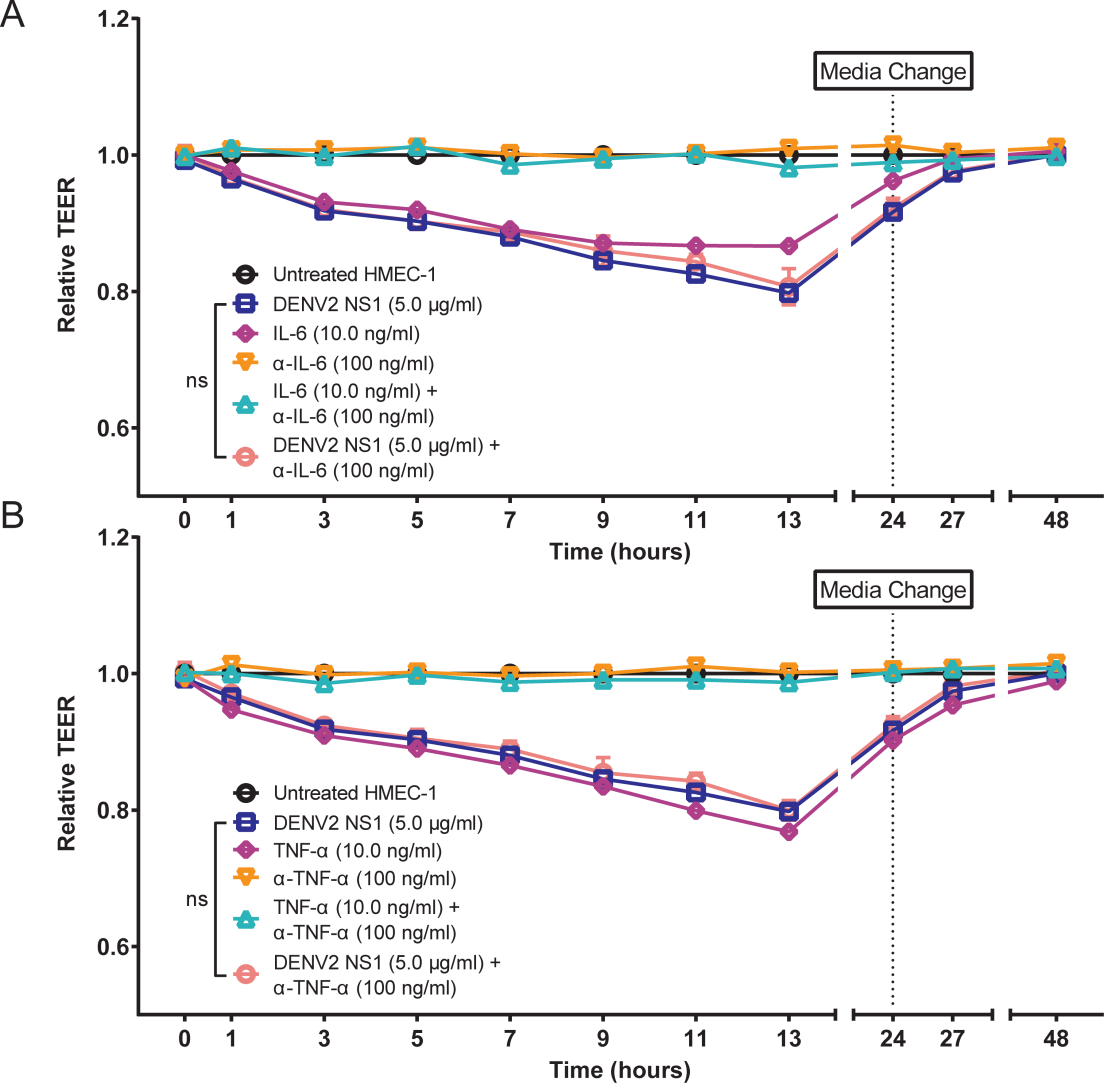
Inflammatory cytokines TNF-α and IL-6 are not involved in DENV2 NS1-induced endothelial hyperpermeability *in vitro*. **(A-B)** Trans-endothelial electrical resistance (TEER) of HMEC-1 monolayers incubated with 5 μg/ml DENV2 NS1 (blue squares), 10 ng/ml recombinant cytokine (**(A)** IL-6, **(B)** TNF-α; purple diamonds), 100 ng/ml anti-cytokine mAbs (**(A)** IL-6, **(B)** TNF-α; orange triangles), recombinant cytokine + specific mAb (**(A)** IL-6, **(B)** TNF-α; green diamonds), or DENV2 NS1 + specific mAb (**(A)** IL-6, **(B)** TNF-α; red circles). The background signal was subtracted (using TEER values from a blank Transwell), and data were normalized to untreated HMEC-1. All data shown represent the mean +/- SEM and were collected from two independent experiments. Data represent two replicate Transwells per condition. A repeated measure two-way ANOVA was used to determine the significance of anti-cytokine mAbs on DENV2 NS1-induced hyperpermeability in HMEC-1. ns = not significant.

To further confirm that IL-6 and TNF-α are not involved in DENV2 NS1-induced endothelial cell-intrinsic mechanisms of endothelial hyperpermeability, we used a Transwell model that measures trans-endothelial electrical resistance (TEER) to evaluate the effect of anti-cytokine monoclonal antibodies (mAbs) on DENV2 NS1-induced endothelial hyperpermeability in HMEC-1 monolayers. We found that both recombinant human IL-6 and TNF-α significantly induced endothelial hyperpermeability (*P* ≤ 0.0001) and that addition of anti-IL-6 and anti-TNF-α mAbs blocked this effect (*P* ≤ 0.0001); however, anti-IL-6 and anti-TNF-α mAbs did not affect DENV2 NS1-induced endothelial hyperpermeability (*P* = 0.1845, *P* = 0.1879, respectively) **(Fig 3A and 3B)**. Similar results were obtained when evaluating HPMEC as well **(S4 Fig)**. Taken together, these results suggest that IL-6 and TNF-α are not involved in the direct action of DENV2 NS1 on the human endothelium *in vitro*.

### DENV2 NS1-induced vascular leak is independent of TLR4 and TNF-α signaling *in vivo*

**Fig 4:**
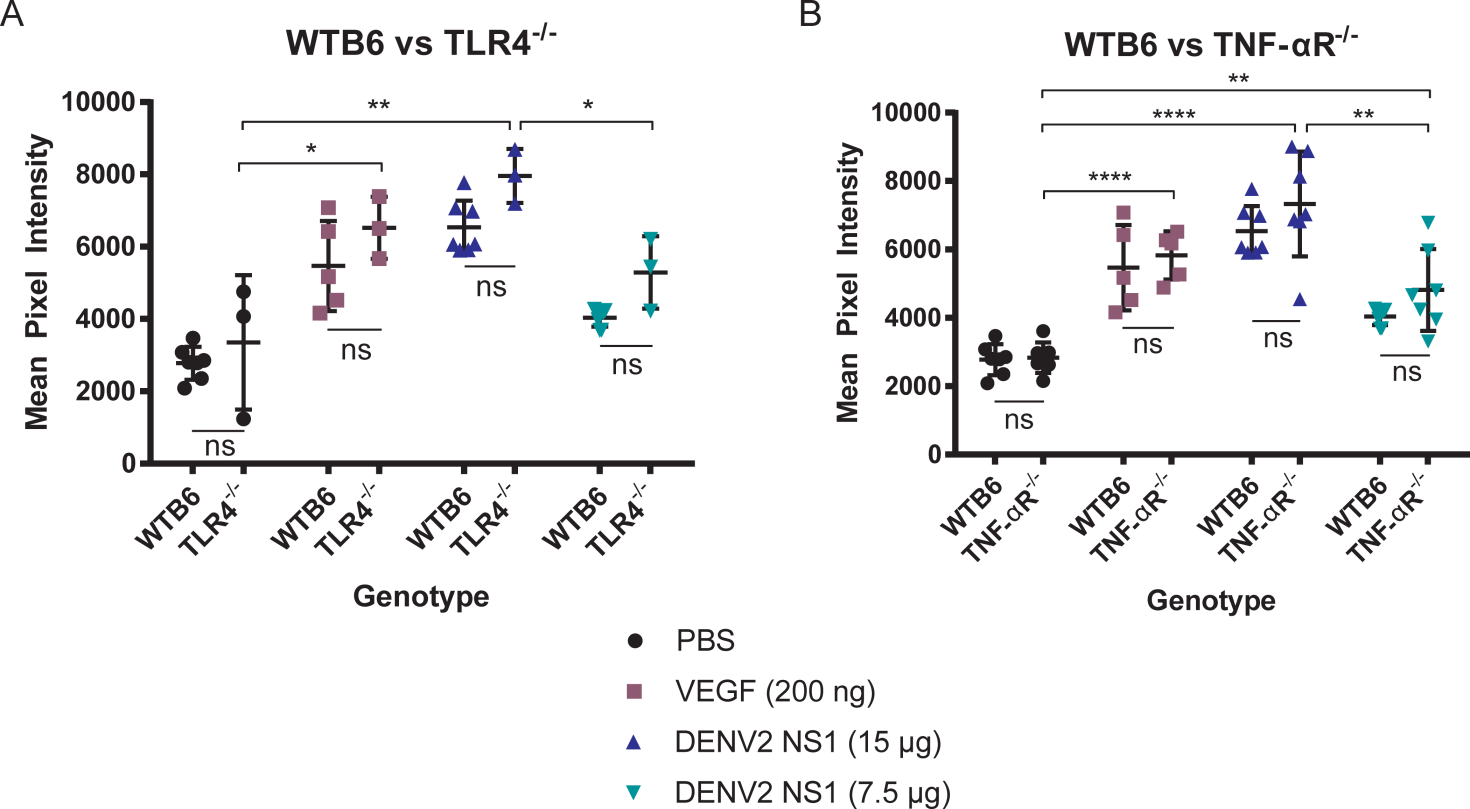
DENV2 NS1-induced vascular leak is independent of TLR4 and TNF-α signaling *in vivo*. **(A-B)** Hair was removed from the dorsal dermis of **(A-B)** wild-type (n = 7), **(A)** *Tlr4*^*-/-*^ (n = 3) and **(B)** *TNF-αR*^*-/-*^ (n = 7) B6 mice, and mice were allowed to recover for 3 days. On the day of the assay, retro-orbital injections of Alexa Fluor 680-conjugated dextran were administered, followed by intradermal injections of PBS (black circles), 200 ng VEGF (purple squares), 15 μg DENV2 NS1 (blue triangles), and 7.5 μg DENV2 NS1 (green triangles). The dermis from each mouse was collected and processed two hours post-injection. Data represent quantification of mean fluorescent intensity from the dermis +/- SEM and were collected from 2–3 independent experiments. The same wild-type B6 mice are used for comparison in both **(A)** and **(B)**. An ordinary one-way ANOVA with multiple comparisons to the PBS group using Dunnett’s multiple comparison test was used to determine significance of VEGF and DENV2 NS1. An unpaired, parametric, two-tailed t-test was used to determine significance between wild-type and knockout mice for each treatment group. ns = not significant, *P < 0.05, **P < 0.01, ****P < 0.0001.

To follow up on our *in vitro* results and to assess the role of TLR4 *in vivo*, we used our murine fluorescent dextran model of dermal vascular leak to evaluate the contributions of TLR4 and TNF-α signaling to DENV2 NS1-induced vascular leak. In both TLR4- and TNF-α receptor (TNF-αR)-deficient B6 mice, we found that both 7.5 μg and 15 μg of DENV2 NS1 triggered vascular leak at similar levels as observed in wild-type B6 mice, though knockout mice demonstrated slightly higher levels of leak than wild-type mice across all conditions **(Fig 4A and 4B)**. To further investigate the role of TLR4 in DENV NS1 pathogenesis, we utilized a model of systemic vascular leak in wild-type and TLR4-deficient B6 mice. Briefly, mice were injected IV with 10 mg/kg of DENV2 NS1 or 10 mg/kg of ovalbumin as a protein control. Three days post-injection, EBD was administered RO and allowed to circulate for 3 hours. Mice were then euthanized, and tissues were harvested for EBD extraction. We found that similar amounts of EBD extravasated into the lungs and liver of TLR4-deficient mice and wild-type B6 mice, though the levels were slightly lower in *Tlr4*^*-/-*^ mice, suggesting comparable levels of NS1-induced vascular leak **(S5 Fig)**. Further, when mice deficient in both TLR4 and interferon-α/β receptor (IFNAR) were infected with DENV2, no significant differences were observed in either morbidity or mortality when compared with IFNAR-deficient B6 mice, though a slight delay in both morbidity and mortality were observed in doubly deficient mice **(S6 Fig)**. These data indicate that TLR4 and TNF-α are not substantially involved in the endothelial cell-specific mechanism of DENV2 NS1-induced vascular leak *in vivo*.

### Inhibition of sialidases, cathepsin L, and heparanase prevents DENV2 NS1-induced vascular leak

**Fig 5:**
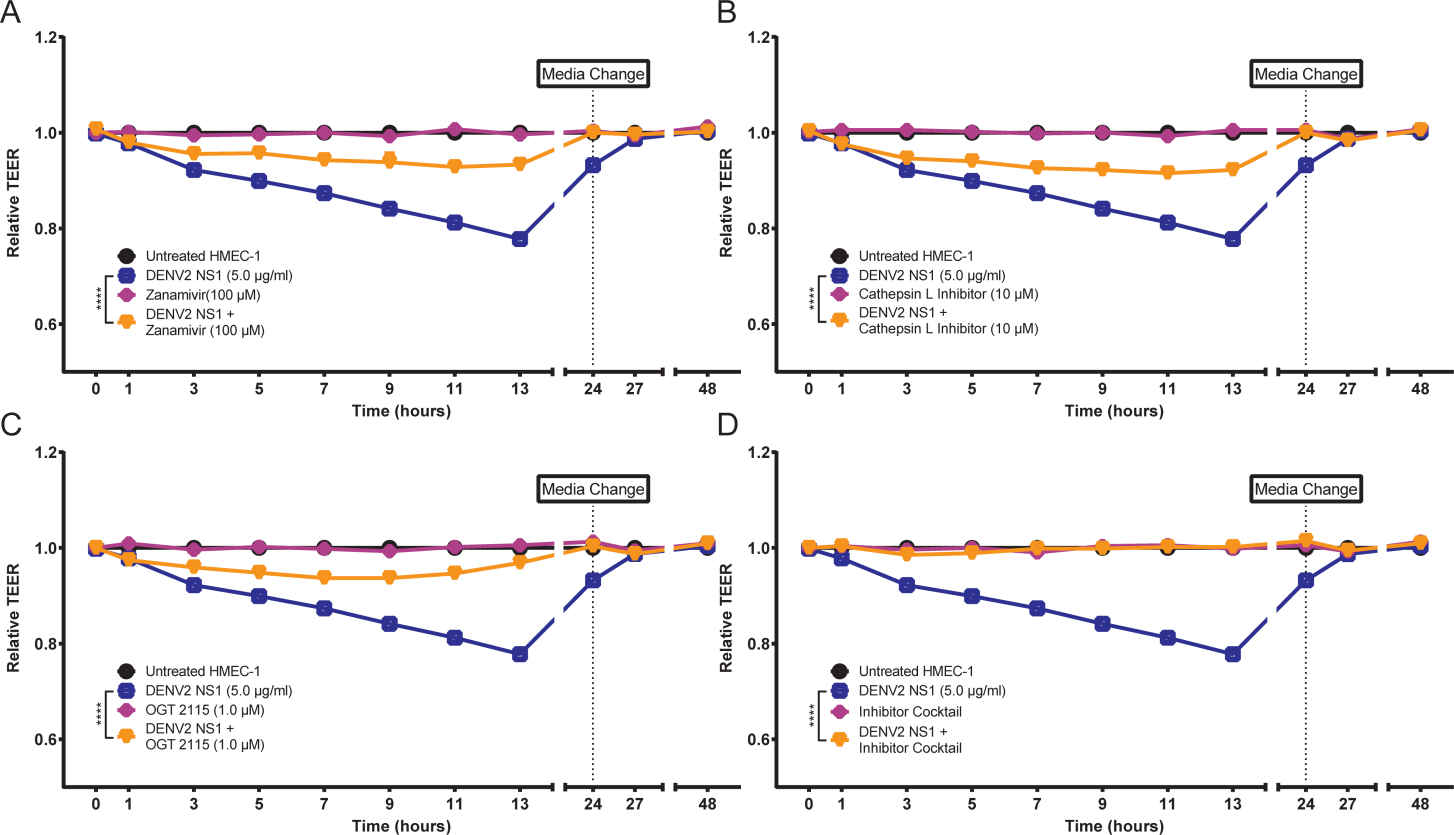
Inhibition of sialidases, cathepsin L, and heparanase prevents DENV2 NS1-induced endothelial hyperpermeability *in vitro*. **(A-D)** Trans-endothelial electrical resistance (TEER) of HMEC-1 monolayers incubated with 5 μg/ml DENV2 NS1 (blue squares), specific inhibitor alone (**(A)** Zanamivir, 100 μM, **(B)** Cathepsin L inhibitor, 10 μM, **(C)** OGT 2115, 1.0 μM, **(D)** inhibitor cocktail; purple diamonds), or DENV2 NS1 + specific inhibitors (**(A)** Zanamivir, 100 μM, **(B)** Cathepsin L inhibitor, 10 μM, **(C)** OGT 2115, 1.0 μM, **(D)** inhibitor cocktail; orange triangles). The background signal was subtracted (using TEER values from a blank Transwell), and data were normalized to untreated HMEC-1. All data shown represent the mean +/- SEM and were collected from two independent experiments with two replicate Transwells per condition. A repeated measure two-way ANOVA was used to determine the significance of anti-cytokine mAbs on DENV2 NS1-induced hyperpermeability in HMEC-1. ****P < 0.0001.

We previously demonstrated that the EGL is an important determinant of endothelial dysfunction triggered by DENV2 NS1 in HPMEC. Specifically, human sialidases, cathepsin L, and heparanase were implicated as enzymes responsible for degrading the EGL and whose expression and activation were triggered by DENV NS1. Further, we showed that sialic acid was degraded and cathepsin L activity was increased following DENV2 NS1 treatment of HMEC-1 [17]. Here, we utilized the TEER system to evaluate the effect of specific inhibitors that target sialidase (Zanamivir), cathepsin L (Cathepsin L Inhibitor), and heparanase (OGT 2115), on DENV2 NS1-induced endothelial hyperpermeability of HMEC-1 *in vitro*. We found that all three inhibitors partially abrogated the increased permeability observed following treatment with DENV2 NS1 (*P* ≤ 0.0001) (**Fig 5A–5C**). Further, a cocktail of all three inhibitors completely eliminated hyperpermeability *in vitro* (*P* ≤ 0.0001) (**Fig 5D**), reflecting our previous work with HPMEC [17]. These findings demonstrate that endothelial cell-intrinsic enzymes contribute importantly to DENV2 NS1-induced endothelial hyperpermeability *in vitro*.

**Fig 6:**
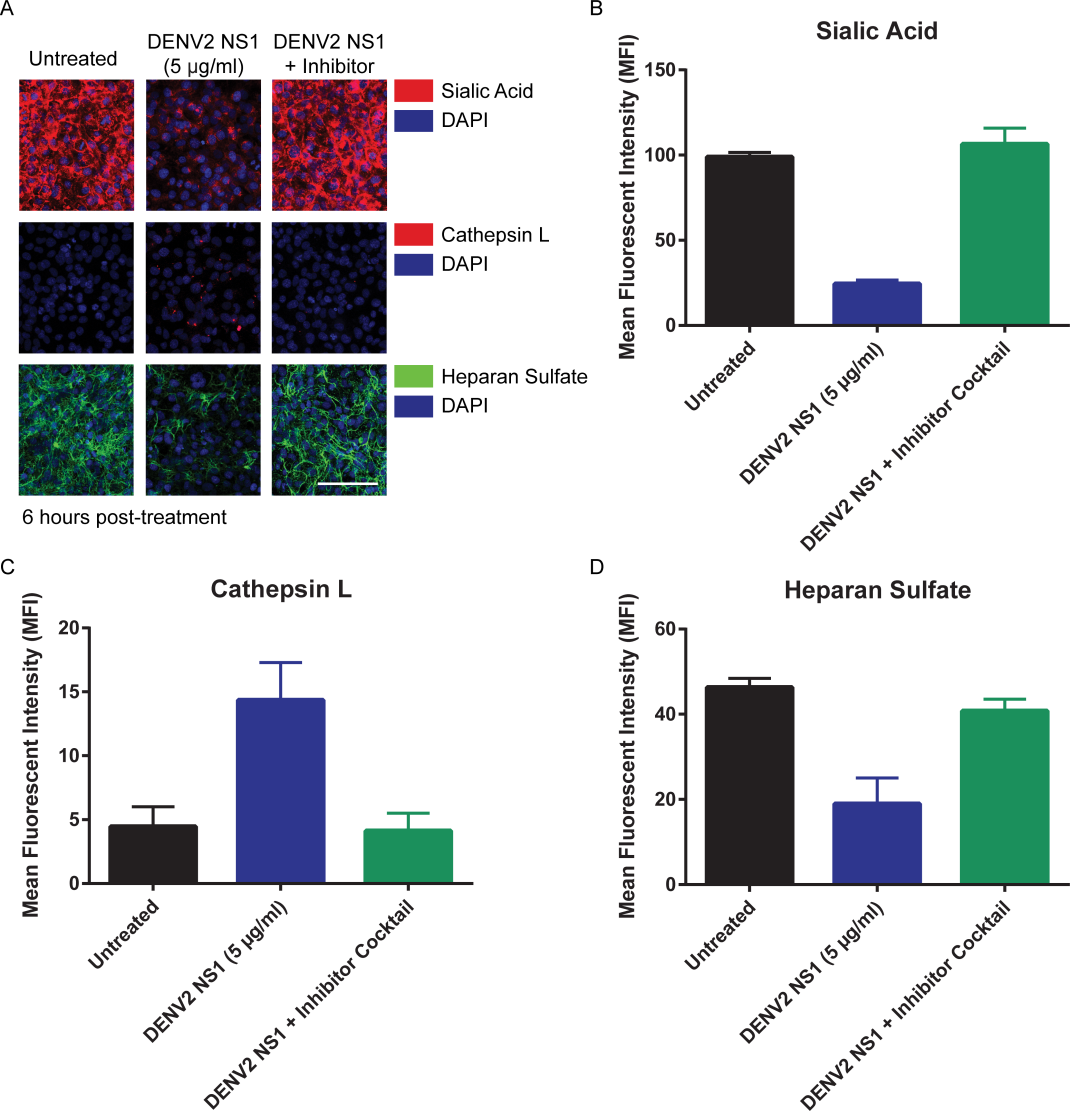
DENV2 NS1 induces degradation of sialic acid, activation of cathepsin L, and shedding of heparan sulfate in HMEC-1 *in vitro*. **(A-D)** HMEC-1 monolayers treated with 5 μg/ml of DENV2 NS1 (middle column) or 5 μg/ml of DENV2 NS1 and an inhibitor cocktail (Zanamivir, 100 μM; Cathepsin L Inhibitor, 10 μM; OGT 2115, 1.0 μM; right column). Untreated monolayers were used as a control (left column). Six hours post-treatment, cells were stained for **(B)** sialic acid (WGA-A647, red; top row images), **(C)** cathepsin L activity (Magic Red Cathepsin L detection kit, red; middle row images), or **(D)** heparan sulfate (Heparan Sulfate mAb clone F58-10E4, green; bottom row images) and imaged on a Zeiss LSM 710 Axio Observer inverted fluorescence microscope equipped with a 34-channel spectral detector at 20x magnification. **(A)** Images were acquired using the Zen 2010 software (Zeiss). Nuclei were stained with *Hoechst* (blue). Images shown at 20X; scale bar, 10 μM. Representative images shown. **(B-D)** Quantification of MFI in **Fig 6A**.

Additionally, we performed confocal microscopy on HMEC-1 monolayers to assess the presence of glycocalyx components, including sialic acid, chondroitin sulfate, heparan sulfate, and hyaluronic acid, and found that all components were expressed at high levels on the surface of HMEC-1 **(S7 Fig)**. Further, we found that DENV2 NS1 induced the degradation of sialic acid, the upregulation of cathepsin L activity, and the shedding of heparan sulfate in HMEC-1 at 6 hours post-treatment, and these effects could be prevented through the use of the previously-mentioned inhibitor cocktail **(Fig 6)**. These data provide additional support that HMEC-1 express an EGL composed of known glycocalyx components.

**Fig 7:**
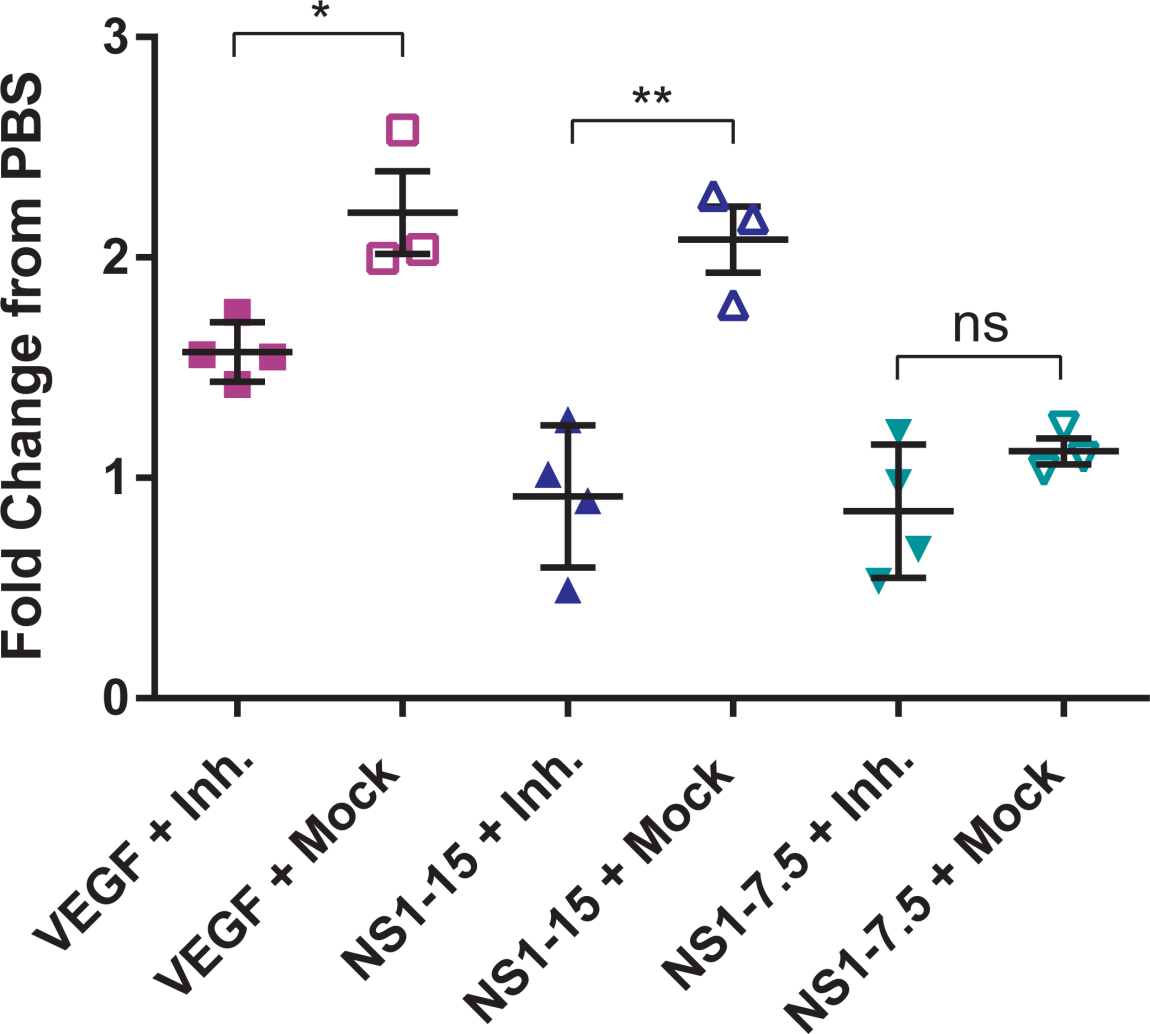
Inhibition of sialidases, cathepsin L, and heparanase prevents DENV2 NS1-induced endothelial hyperpermeability *in vivo*. Hair was removed from the dorsal dermis of wild-type B6 mice, and mice were allowed to recover for 3 days. On the day of the assay, mice received an intraperitoneal dose of inhibitor cocktail (Zanamivir, Cathepsin L Inhibitor, and OGT 2115; 1 mg/ml of each inhibitor) 6 hours pre-assay and then immediately preceding the start of the assay (n = 4; closed symbols). Control mice received injections of DMSO, PBS, and water as a vehicle control (n = 3; open symbols). Retro-orbital injections of Alexa Fluor 680-conjugated dextran were then administered, followed by intradermal injections of PBS (black circles), 200 ng VEGF (purple squares), 15 μg DENV2 NS1 (blue triangles), and 7.5 μg DENV2 NS1 (green triangles). The dermis from each mouse was collected and processed two hours post-injection. Data represent the fold change of mean fluorescent intensity from VEGF and DENV2 NS1 injections to PBS injections. Data represent mean +/- SEM and were collected from 2 independent experiments. Unpaired, parametric, two-tailed t-tests were used to determine significance between inhibitor-treated and mock-treated groups. ns = not significant, *P < 0.05, **P < 0.01.

Next, we sought to determine the relative contribution of glycocalyx components to vascular leak *in vivo*. Wild-type B6 mice were administered an intraperitoneal (IP) dose of a cocktail containing the same sialidase, cathepsin L, and heparanase inhibitors used *in vitro* 6 hours before and immediately preceding ID injections of DENV2 NS1. Experiments were then performed as previously detailed. A separate group of mice was administered a combination of DMSO, PBS, and water as a vehicle control. We found that mice receiving the inhibitor cocktail as opposed to the vehicle control demonstrated a significantly lower fold-change in vascular leak as compared to PBS when exposed to 15 μg of DENV2 NS1 (inhibitor cocktail: 0.917; vehicle control: 2.08; *P* = 0.0038), and the data trended towards a decrease in fold-change when exposed to 7.5 μg of NS1 (inhibitor cocktail: 0.848; vehicle control: 1.12) (**Fig 7**). VEGF-induced vascular leak was also decreased with the inhibitor cocktail but not as strongly as DENV2 NS1 (inhibitor cocktail: 1.57; vehicle control: 2.20; *P* = 0.0156). Taken together, these data demonstrate that endothelial sialidases, cathepsin L, and heparanase strongly contribute to the local vascular leak induced by DENV2 NS1 in the dermis of wild-type B6 mice.

## Discussion

In this study, we demonstrate that DENV2 NS1 can directly trigger vascular leak in the dermis of wild-type B6 mice in the absence of viral infection and that this effect is specific to DENV2 NS1 and was not observed with WNV NS1. *In vivo*, TLR4 and TNF-α did not contribute to NS1-induced vascular leak in our dermal model, which reflects endothelial cell-intrinsic NS1-triggered leak. Consistent with this observation, human dermal endothelial cells did not produce TNF-α or IL-6 in response to DENV2 NS1 stimulation *in vitro*, nor was NS1-induced endothelial hyperpermeability mediated by these cytokines. Finally, we show that inhibition of endothelial sialidases, cathepsin L, and heparanase is sufficient to prevent vascular leak induction by DENV2 NS1 both *in vitro* and *in vivo*.

Endothelial glycocalyx components have not previously been experimentally shown to play a role in dengue pathogenesis *in vivo*. Here, we showed that disruption of glycocalyx components is responsible for the induction of vascular leak observed in the murine dermis by DENV2 NS1. We were able to protect mice from local vascular leak using a combination of Zanamivir, Cathepsin L Inhibitor, and OGT 2115, demonstrating that the activity of endothelial sialidases, cathepsin L, and heparanase mediate the disruption of endothelial barrier function by DENV2 NS1 *in vivo*. These enzymes contribute to glycocalyx disruption via degradation of sialic acid and trimming of heparan sulfate proteoglycans. Using our *in vitro* HMEC-1 model of endothelial permeability, we demonstrated that inhibition of each enzyme alone partially abrogated NS1-induced hyperpermeability, while the combination of all three inhibitors completely protected endothelial monolayers. Our results are consistent with previous findings that demonstrated that NS1 induces endothelial hyperpermeability by increasing expression of sialidases, cathepsin L, and heparanase in human pulmonary endothelial cells *in vitro* [17]. These results suggest potential targets for novel therapeutics that inhibit enzymes that contribute to degradation of glycocalyx components for the treatment of severe dengue disease.

It has previously been shown that TLR4 is an important mediator of DENV NS1 pathogenesis, as NS1 activation of TLR4 on PBMCs leads to production of IL-6 and IL-8, cytokines that have been shown to contribute to endothelial barrier dysfunction [20]. Therefore, we evaluated the effect of DENV2 NS1 on local and systemic vascular leak in TLR4-deficient B6 mice and found no significant differences in the levels of EBD and dextran extravasation in TLR4-deficient and wild-type mice following NS1 inoculation, suggesting that NS1 induces acute vascular leak that is not dependent on TLR4. In addition, infection of mice doubly deficient in TLR4 and IFNAR with a lethal dose of DENV2 did not result in significant differences in morbidity or mortality compared to IFNAR-deficient mice. However, slight differences were observed between wild-type and TLR4-deficient mice in all assays. In our model of localized vascular leak, levels of leak were marginally higher in *Tlr4*^*-/-*^ mice, whereas levels were slightly lower in our systemic vascular leak model. In our DENV infection model, morbidity and mortality were both somewhat delayed in *Ifnar*^*-/-*^ *x Tlr4*^*-/-*^ B6 mice, though mice succumbed to infection at the same rates. Modhiran et al. found that LPS-RS, a potent antagonist of TLR4, and an anti-TLR4 antibody both protected HMEC-1 monolayers from DENV2 NS1-induced endothelial hyperpermeability as measured by TEER [20]. These observations suggest that TLR4 does play a contributing role to NS1-induced vascular leak, but because substantial vascular leak, morbidity, and mortality are still observed in the absence of TLR4, we conclude there are additional, more critical drivers of NS1 pathogenesis.

Previously, we demonstrated that systemic administration of DENV2 NS1 resulted in significantly elevated levels of IL-6 and TNF-α 72 hours post-injection [16]. We sought to evaluate whether endothelial cells produce these inflammatory cytokines in response to DENV2 NS1 in the absence of PBMCs. We demonstrated that HMEC-1 did not produce IL-6, TNF-α, or IL-8 in response to DENV2 NS1, although HMEC-1 produced high levels of IL-6 and IL-8 in response to the positive control, the TLR4 ligand LPS. Further, to evaluate the role of these cytokines in our HMEC-1 model of endothelial permeability, we measured TEER in the presence of both DENV2 NS1 and IL-6- and TNF-α-specific mAbs. We demonstrated that DENV2 NS1-induced endothelial hyperpermeability is independent of both IL-6 and TNF-α, although HMEC-1 can respond to both cytokines, an effect that is prevented with blocking antibodies. We then sought to determine the contribution of TNF-α to NS1-induced vascular leak *in vivo*. Using TNF-αR-deficient B6 mice, we found that DENV2 NS1 triggered similar levels of vascular leak in both knockout and wild-type mice. These results suggest that NS1 stimulation of endothelial cells does not result in the production of pro-inflammatory cytokines and that any directly pathogenic effects of NS1 on the endothelium occur independently of key endothelial cell-produced cytokines, further emphasizing the role of endothelial cell-specific mechanisms such as glycocalyx component disruption and mislocalization of intercellular junction proteins.

In humans, severe symptoms of vascular leak appear several days after the peak of NS1 antigenemia in blood has passed, suggesting a requirement for prolonged exposure to NS1 to cause severe leak and presumably reflecting the cumulative effects and accumulation of NS1 in target tissues. Our previous mouse model data, also requiring three days of exposure to NS1 delivered systemically before morbidity and mortality were observed [16], suggest that the induction of systemic vascular leak caused by NS1 is cumulative over time *in vivo*. The NS1 dose that we used here in our *in vivo* model of localized vascular leak was meant to reflect the cumulative effect of NS1 in tissues, allowing us to simulate the effects of NS1 on the endothelium in a shorter time span. Overall, the endothelial-intrinsic mechanisms we observe following NS1 inoculation in the dermis may be working in conjunction with NS1 activation of TLR4 on the surface of PBMCs and release of inflammatory cytokines to contribute to systemic vascular leak observed in both our previous findings and clinical manifestations of DHF and DSS.

Clinically, DHF and DSS are both characterized by vascular leak and endothelial dysfunction in multiple organs. The lungs are particularly affected, with pulmonary edema representing a common complication in severe dengue disease. We have previously published a study identifying the EGL as a required determinant of endothelial barrier function in human pulmonary microvascular endothelial cells *in vitro* [17], and our *in vitro* results here further support that DENV2 NS1 triggers endothelial hyperpermeability in HPMEC in a cytokine-independent manner in the absence of virus and non-endothelial cell types. Further, petechiae are an additional sign of capillary fragility and leak in dengue disease in humans, and previous reports have shown that DENV is absent at the site of rash and petechiae in dengue fever patients [22], though DENV antigens have been found in skin biopsies [23]. Here, we demonstrated the ability of DENV2 NS1 to trigger localized vascular leak in the absence of virus in the mouse dermis, suggesting a potential link between DENV NS1 and formation of petechiae. Combined with our results in HPMEC, our findings suggest that NS1 may be an important contributor to vascular leak in various tissue sites during DENV disease.

The glycocalyx has been previously implicated in plasma leakage in DENV-infected patients. Nguyen-Pouplin et al. performed dextran fractional clearance studies in a group of Vietnamese dengue patients with evidence of vascular leak and in healthy controls and found that there was no difference in dextran clearance between infected and healthy patients, seemingly contradictory results. However, their data suggest that the dextran administered for the study may have helped stabilize the glycocalyx following loss of plasma proteins during DENV infection, thereby restoring glycocalyx integrity and normal endothelial barrier function [10]. More recent studies have evaluated different glycocalyx components in patient sera during acute DENV infection and found that serum levels of hyaluronic acid, heparan sulfate, chondroitin sulfate, and syndecan-1 are all elevated, suggesting disruption of the endothelial glycocalyx by DENV infection [18, 19].

Ascertainment that disruption of the endothelial glycocalyx is mediated by DENV NS1 entails direct visualization of the glycocalyx layer *in vivo*. As the glycocalyx is a delicate and complex structure, this direct visualization requires challenging techniques, such as intravital microscopy, two-photon microscopy, and electron microscopy, which have previously been used to image the glycocalyx in live mice and in fixed murine tissues [24–27]. Further, though cultured endothelial cells express key components of the glycocalyx on their surface, they do not necessarily express a true glycocalyx structure *in vitro*. Thus, drawing definitive conclusions regarding the glycocalyx and its role in dengue disease is difficult; however, we believe our results support a critical role for glycocalyx components and thus implicitly for the glycocalyx in vascular leak, though further validation *in vivo* is necessary.

As we have previously speculated, we believe that DENV NS1 induces endothelial hyperpermeability following its internalization by endothelial cells [17]. After NS1 binds to an as-yet unidentified receptor, internalization may then facilitate NS1 interaction with cathepsin L (which can then cleave heparanase into an active form) and the endothelial sialidase Neu1, both of which are localized in lysosomal compartments [28, 29]. It is also possible that binding and downstream signaling triggers activation of these enzymes, specifically Neu3, which is localized to the plasma membrane [30]. Preliminary data from our laboratory suggests that inhibition of endocytic pathways in endothelial cells may protect monolayers *in vitro* from DENV NS1-induced hyperpermeability. Further research is required to fully elucidate the mechanisms driving DENV NS1-related pathogenesis.

Finally, our model improves upon the existing Miles assay that uses Evans Blue dye as a tracer for vascular leak. By using dextran conjugated to Alexa Fluor 680, we were able to observe greater differences between groups in our model, as well as decreased variability as compared to using EBD. Additionally, we are able to evaluate positive and negative controls alongside experimental conditions in the same animal. These factors serve to decrease the total number of mice required to achieve statistical significance of our data, saving on costs and contributing to the overall goal of limiting animal numbers used in experiments. Additionally, this method does not require excision of individual spots or extraction of dye but allows for direct imaging of the skin on a fluorescent scanner, thereby decreasing the time spent processing each animal and allowing for faster development and analysis of results. Compared to our systemic models of vascular leak [16], the modified Miles assay facilitates the evaluation of the direct and immediate effects of NS1 on the vascular endothelium, as ID injections deliver the protein to a localized spot. Additionally, this allows for usage of substantially lower quantities of NS1 protein. It is, however, possible that compounds delivered intradermally may interact with resident dermal immune cells or the abluminal surface of the endothelium following inoculation. Thus, results obtained in the intradermal assay should be validated using intravenous delivery of NS1 in a systemic model of leak, as we have done here.

Taken together, these results contribute to the growing body of literature demonstrating a critical role for NS1 in dengue pathogenesis, specifically in its contributions to directly pathogenic effects on the vascular endothelium. We show that DENV2 NS1-induced endothelial barrier dysfunction is not related to inflammatory cytokines in a mouse model of dermal vascular leak or in endothelial cells *in vitro* but that instead, specific endothelial enzymes that degrade components of the endothelial glycocalyx layer are required. Overall, these findings further illustrate the role of NS1 in the pathogenesis of severe dengue disease and highlight the importance of the endothelial glycocalyx in vascular leak, suggesting targets for novel therapeutics in the treatment of DHF and DSS.

## Materials and methods

### Ethics statement

All *in vivo* experiments were performed strictly following the guidelines of the American Veterinary Medical Association and the Guide for the Care and Use of Laboratory Animals from the National Institutes of Health and were approved by the University of California (UC) Berkeley Animal Care and Use Committee (protocol AUP-2014-08-6638).

### Mice

Six-to-eight-week-old wild-type C57BL/6 (B6) mice were obtained from the Jackson Laboratory. *Tlr4*^*-/-*^ and *TNF-αR*^*-/-*^ B6 mice were originally obtained from Dr. Greg Barton (UC Berkeley) and the Jackson Laboratory, respectively. *Ifnar*^*-/-*^ B6 were originally obtained from the Jackson Laboratory. *Tlr4*^*-/-*^ *x Ifnar*^*-/-*^ B6 mice were generated at UC Berkeley by backcrossing *Tlr4*^*-/-*^ onto an *Ifnar*^*-/-*^ B6 background 10 times. All mice were bred and maintained in specific pathogen-free conditions at the animal facility at UC Berkeley. A mix of male and female six-to-eight-week-old mice were used in all experiments. Trained and certified laboratory personnel performed anesthesia of mice via isoflurane inhalation and euthanasia of mice using exposure to isoflurane followed by cervical dislocation.

### Cell culture and viruses

The human dermal microvascular endothelial cell line HMEC-1 was kindly donated by Dr. Matthew Welch (UC Berkeley) and propagated (passages 18–25) and maintained at 37°C in humidified air with 5% CO_2_ in MCDB 131 medium (Sigma) supplemented with 1% penicillin/streptomycin (Life Technologies), 0.2% Epidermal Growth Factor (Life Technologies), 0.4% hydrocortisone (Sigma), and 5% Fetal Bovine Serum (Corning). The human pulmonary microvascular endothelial cell line HPMEC-ST1.6r (HPMEC) was kindly donated by Dr. J.C. Kirkpatrick (Institute of Pathology, Johannes Gutenberg University, Germany) and grown as previously described [17]. DENV2 D220 was generated in our laboratory from the parental strain DENV2 PL046 [31]. Virus was propagated in the *Aedes albopictus* C6/36 cell line (American Type Culture Collection; ATCC) and titered by plaque assay on baby hamster kidney cells (BHK21, clone 15).

### Recombinant NS1 proteins

Recombinant DENV1 (Nauru/Western Pacific/1974), DENV2 (Thailand/16681/84), DENV3 (Sri Lanka D3/H/IMTSSA-SRI/2000/1266), DENV4 (Dominica/814669/1981), and WNV (NY99) NS1 proteins, greater than 95% purity and certified to be free of endotoxin contaminants, were produced by the Native Antigen Company (Oxfordshire, United Kingdom) in HEK293 cells at their facility and used in all experiments. NS1 preparations were also tested using the Endpoint Chromogenic Limulus Amebocyte Lysate (LAL) QCL-1000 kit (Lonza) and confirmed to be free of bacterial endotoxins [17].

### Recombinant proteins, monoclonal antibodies, and inhibitors

For staining of EGL components, the following monoclonal antibodies and lectins were used: Wheat germ agglutinin (WGA) lectin conjugated to Alexa Fluor 647 (WGA-A647, Molecular Probes) to stain N-acetyl neuraminic acid (sialic acid); Ab Heparan Sulfate, purified (clone F58-10E4, Amsbio); anti-Chondroitin Sulfate antibody (CS-56, Abcam); anti-Hyaluronic acid antibody (Abcam). Goat anti-mouse IgG conjugated to Alexa Fluor 647 (Abcam), donkey anti-mouse IgM conjugated to Alexa Fluor 488 (Jackson), and donkey anti-sheep IgG conjugated to Alexa Fluor 568 (Abcam) were used as secondary detection antibodies in confocal microscopy experiments. Vascular endothelial growth factor (VEGF; Sigma) was used as a positive control in *in vivo* dermal Miles assay experiments. Ovalbumin (Life Technologies) was used as a negative control in *in vivo* systemic Miles assay experiments. Anti-Flavivirus group antigen against the DENV envelope (E) protein (4G2, clone number D1-4G2-4-15; Absolute Antibody) was used in antibody-dependent enhancement (ADE) infections *in vivo*. Recombinant human TNF-α and recombinant human IL-6 (eBiosciences) were used in trans-endothelial electrical resistance (TEER) assays. Anti-human TNF-α and anti-human IL-6 mAbs (eBiosciences; clones MAb1 and MQ2-13A5, respectively) were used in anti-cytokine and anti-NS1 TEER experiments. Selective inhibitors of sialidases (Zanamivir, Sigma), heparanase (OGT 2115, Tocris), and cathepsin L (Cathepsin L Inhibitor, Santa Cruz Biotechnology) were used in TEER assays and in mouse experiments at concentrations that do not affect cell viability, animal welfare, or vascular leakage. Cell viability was determined using the Promega CellTox Green Cytotoxicity Assay following the manufacturer’s instructions.

### Evans Blue dermal Miles assay

The effect of recombinant DENV2 NS1 protein on localized vascular leak *in vivo* was evaluated using the Miles assay adapted for mouse skin as previously described [32]. Wild-type B6 mice were shaved 3–4 days prior to each experiment using Wahl Show Pro Plus clippers, and hair was further removed using Nair (Church & Dwight) and 70% ethanol. On the day of the assay, mice were anesthetized with isoflurane and injected RO with EBD (0.5% in PBS, 150 μl; Sigma). After 10 minutes, PBS (50 μl), VEGF (200 ng in 50 μl PBS), and DENV2 NS1 (15 μg or 7.5 μg in 50 μl PBS) were injected ID into distinct sites on the shaved dorsal skin of mice. Two hours post-injection, mice were euthanized using isoflurane, and the dorsal dermis was removed. A 13 mm diameter circular punch was used to mark the biopsy site surrounding the locations of Evans Blue leakage, and sites were removed using a surgical scalpel and placed in formamide for 24 hours at 56°C. The absorbance of the extravasated dye was measured at 620 nm using a spectrophotometer. EBD concentration was calculated using a standard curve (30.5 to 500,000 ng/ml) using linear regression analysis. Representative images were obtained using an iPhone 5S (Apple).

### Dextran-adapted dermal Miles assay

Dorsal hair was removed from mice as described above. On the day of the assay, mice were anesthetized with isoflurane and injected ID with PBS (50 μl), VEGF (200 ng in 50 μl PBS), and DENV2 NS1 (7.5 μg or 15 μg in 50 μl PBS) and/or WNV NS1 (15 μg in 50 μl PBS) into the shaved back skin of the mouse. Immediately following ID injections, 200 μl of 10 kDa dextran conjugated with Alexa Fluor 680 (1 mg/ml; Sigma) was delivered by RO injection. Two hours post-injection, mice were euthanized using isoflurane, and the dorsal dermis was removed and placed in Petri dishes. Tissues were scanned using a fluorescent detection system (LI-COR Odyssey CLx Imaging System) at a wavelength of 700 nm, and leakage in a 13 mm diameter circle surrounding the sites of injection was quantified using Image Studio software (LI-COR Biosciences). For experiments using inhibitors, compounds were administered IP in a total volume of 200 μl 6 hours before the start of the assay and then immediately before beginning the assay. All inhibitors were used at a final concentration of 1 mg/kg. A combination of DMSO, PBS, and water was used as a vehicle control.

### Intradermal injection of murine ears

Vascular leak in murine ears was measured as previously described [33]. Ears of isoflurane-anesthetized mice were immobilized using cover slip forceps. A sterilized needle (30-gauge, 25 mm length, and 10°-12° bevel), used with a 25 μl reusable glass microinjection syringe (Hamilton), was inserted ~3 mm into the ventral side of the ear skin at a flat angle with the bevel pointing up, and either 20 μl PBS or 7.5 μg DENV2 NS1 diluted in 20 μl PBS was slowly injected. EBD (0.5% in PBS, 150 μl) or 200 μl of 10 kDa dextran conjugated with Alexa Fluor 680 (1 mg/ml) was immediately delivered by RO injection. EBD and dextran were allowed to circulate for 30 minutes and 2 hours, respectively, and mice were then euthanized using isoflurane. For EBD assays, representative images were obtained using an iPhone 5S (Apple). For dextran assays, ears were removed and imaged using a fluorescent detection system (LI-COR Odyssey CLx Imaging System) at a wavelength of 700 nm.

### Evans Blue systemic Miles assay

The effect of recombinant DENV2 NS1 protein on systemic vascular leak *in vivo* was evaluated using the Miles assay as previously described [16]. Briefly, wild-type or *Tlr4*^*-/-*^ B6 mice were anesthetized using isoflurane and injected IV with either 10 mg/kg of ovalbumin or DENV2 NS1. Three days post-injection, mice were administered 200 μl of 0.5% EBD, and dye was allowed to circulate for 3 hours before mice were euthanized and cardiac puncture was performed. Tissues were collected and thoroughly dried in pre-weighed tubes. One ml of formamide was then added and incubated at 56°C for 48 hours. The absorbance of the extravasated dye was measured at 620 nm using a spectrophotometer. EBD concentration was calculated using a standard curve (30.5 to 500,000 ng/ml) using linear regression analysis.

### DENV2 infection of mice

Mice deficient in the interferon-α/β receptor (*Ifnar*^*-/-*^*)* or *Tlr4*^*-/-*^ *x Ifnar*^*-/- *^doubly deficient B6 mice were challenged IV with 10^7^ plaque-forming units (PFU) of DENV2 D220 or 5 μg of 4G2 (anti-DENV E mAb) 20–24 hours prior to infection with 3 x 10^5^ PFU of D220 (ADE). Animals were monitored daily and DENV-induced morbidity and mortality were scored using a standardized scale [34].

### Trans-endothelial electrical resistance (TEER)

The effect of recombinant DENV2 NS1 protein on endothelial permeability was evaluated by measuring TEER in HMEC-1 grown on a 24-well Transwell polycarbonate membrane system (Transwell permeable support, 0.4 μM, 6.5 mm insert; Corning Inc.) as previously described [16, 17]. Briefly, TEER was measured in Ohms (Ω) at sequential 2-hour time-points following the addition of test proteins using an Epithelial Volt Ohm Meter (EVOM) with “chopstick” electrodes (World Precision Instruments). Untreated endothelial cells grown on Transwell inserts were used as negative untreated controls, and inserts with medium alone were used for blank resistance measurements. Relative TEER represents a ratio of resistance values (Ω) as follows: (Ω experimental condition—Ω medium alone) / (Ω non-treated endothelial cells—Ω medium alone). After 24 hours of treatment, 50% of upper and lower chamber media was replaced by fresh endothelial cell medium. For experiments using mAbs, antibodies were added immediately before the addition of test proteins. For experiments using inhibitors, compounds were added to the apical compartment of the Transwell 1 hour before the addition of DENV NS1 protein.

### Fluorescence microscopy

Microscopy was performed as previously described [17]. For imaging experiments, HMEC-1 were grown on coverslips coated with 0.2% gelatin (Sigma) and imaged on a Zeiss LSM 710 Axio Observer inverted fluorescence microscope equipped with a 34-channel spectral detector. Images acquired using the Zen 2010 software (Zeiss) were processed and analyzed with ImageJ software [35]. All RGB images were converted to grayscale, then mean grayscale values and integrated density from selected areas were taken, along with adjacent background readings, and plotted as mean fluorescence intensity (MFI). To assess the effect of DENV2 NS1 on integrity of the EGL architecture, the distribution of sialic acid and heparan sulfate, as well as cathepsin L activity, was examined on untreated confluent HMEC-1 monolayers and on monolayers treated with DENV2 NS1 proteins (5 μg/ml) and fixed with 4% paraformaldehyde (PFA) at 6 hours post-treatment. Confluent untreated HMEC-1 monolayers were also fixed and stained for chondroitin sulfate and hyaluronic acid, normal constituents of the endothelial glycocalyx. Primary antibodies were incubated overnight at 4°C, and detection was performed using secondary species-specific anti-IgG or anti-IgM antibodies conjugated to Alexa fluorophores (488, 568 and 647).

### Enzymatic activity assays

Cathepsin L activity in living cells was monitored using the Magic Red Cathepsin L detection kit (Immunochemistry Technologies, Inc.) as previously described [17]. Briefly, confluent HMEC-1 monolayers grown on coverslips were exposed to DENV2 NS1 protein (5 μg/ml), and at 6 hours post-treatment, a cell membrane-permeant fluorogenic substrate MR-(Phe-Arg)_2_, which contains the cresyl violet (CV) fluorophore branded as Magic Red (MR), was added. Cultured cell monolayers expressing active cathepsin L catalyze the hydrolysis of the two Phe-Arg target sequences, generating a red fluorescent species that can be detected by immunofluorescence microscopy. Magic Red excites at 540–590 nm (590 nm optimal) and emits at >610 nm (630 nm optimal).

### ELISA

TNF-α, IL-6, and IL-8 levels were measured using ELISA assays following the manufacturer’s instructions (Abcam).

### Statistics

Statistical analyses were performed using GraphPad Prism 6 software, and all graphs were generated using Prism 6. For *in vivo* murine dermis experiments, an ordinary one-way ANOVA with multiple comparisons to the PBS group using Dunnett’s multiple comparison test was used to determine significance of VEGF, DENV2 NS1, and WNV NS1. An unpaired, parametric, two-tailed t-test was used to determine significance between individual groups. For DENV infection experiments, comparison of survival rates was conducted using a nonparametric log-rank (Mantel-Cox) test and graphed as Kaplan-Meier survival curves. For ELISA experiments, a repeated measure two-way ANOVA with multiple comparisons to the untreated group using Dunnett’s multiple comparison test was used to determine significance of treatment with LPS (10 and 100 ng/ml) or DENV2 NS1 (5 and 10 μg/ml). For TEER experiments, a repeated measure two-way ANOVA was used to determine the significance of treatments (i.e. anti-cytokine mAbs or inhibitors) on DENV2 NS1-induced hyperpermeability in HMEC-1. Significance was further confirmed using a repeated measure two-way ANOVA with multiple comparisons to the untreated group using Dunnett’s multiple comparison test as well as an ordinary one-way ANOVA with multiple comparisons to the PBS group using Dunnett’s multiple comparison test of area under the curve values for each group.

## Supporting information

S1 Fig Related to Fig 1. **NS1 from DENV1-4 triggers localized vascular leak in the dorsal dermis of mice.** Representative image of mouse dorsal dermis following fluorescent dextran assay. Hair was removed from the dorsal dermis of mice, and mice were allowed to recover for 3 days. On the day of the assay, retro-orbital injections of Alexa Fluor 680-conjugated dextran were administered, followed by intradermal injections of PBS, 15 μg DENV1 NS1, 15 μg DENV2 NS1, 15 μg DENV3 NS1, and 15 μg DENV4 NS1. The dermis from each mouse was collected and processed two hours post-injection and scanned using a fluorescent detection system (LI-COR Odyssey CLx Imaging System) at a wavelength of 700 nm, and images were obtained using Image Studio software (LI-COR Biosciences).
(TIF)

S2 Fig Related to Fig 1. **DENV2 NS1 triggers localized vascular leak in the dermis of mouse ears. (A-B)** Wild-type B6 mice received intradermal injections with either PBS (right ear) or 7.5 μg DENV2 NS1 (left ear) and, immediately after, intravenous injections of either **(A)** Evans Blue dye (EBD) or **(B)** Alexa Fluor 680-conjugated dextran. **(A)** EBD was allowed to circulate for 30 minutes and ears were photographed. **(B)** Dextran was allowed to circulate for 2 hours. Ears were removed and scanned using a fluorescent detection system (LI-COR Odyssey CLx Imaging System) at a wavelength of 700 nm, and images obtained using Image Studio software (LI-COR Biosciences).
(TIF)

S3 Fig Related to Fig 2. **HPMEC do not produce the inflammatory cytokines IL-6, TNF-α, or IL-8 in response to DENV2 NS1 stimulation *in vitro*. (A-C)** HPMEC were stimulated with LPS (0.1 or 10 μg/ml; red squares and orange triangles, respectively) or DENV2 NS1 (5 or 10 μg/ml; dark blue triangles and light blue diamonds, respectively), and supernatant was collected at 0, 1, 3, 6, and 24 hours post-treatment. Untreated HPMEC monolayers were used as a control (black circles). ELISAs for **(A)** IL-6, **(B)** TNF-α, and **(C)** IL-8 were performed on all samples.
(TIF)

S4 Fig Related to Fig 3. **Inflammatory cytokines TNF-α and IL-6 are not involved in DENV2 NS1-induced endothelial hyperpermeability in HPMEC *in vitro*. (A-B)** Trans-endothelial electrical resistance (TEER) of HPMEC monolayers incubated with 5 μg/ml DENV2 NS1 (blue squares), 5 ng/ml recombinant cytokine (**(A)** IL-6, **(B)** TNF-α; purple diamonds), 50 ng/ml anti-cytokine mAbs (**(A)** IL-6, **(B)** TNF-α; orange triangles), recombinant cytokine + specific mAb (**(A)** IL-6, **(B)** TNF-α; green diamonds), or DENV2 NS1 + specific mAb (**(A)** IL-6, **(B)** TNF-α; red circles). The background signal was subtracted (using TEER values from a blank Transwell), and data were normalized to untreated HPMEC. All data shown represent the mean +/- SEM and were collected from two independent experiments. Data represent two replicate Transwells per condition. A repeated measure two-way ANOVA was used to determine the significance of anti-cytokine mAbs on DENV2 NS1-induced hyperpermeability in HPMEC. ns = not significant, *P < 0.05.
(TIF)

S5 Fig Related to Fig 4. **DENV2 NS1-induced systemic vascular leak *in vivo* is similar in wild-type and TLR4-deficient mice. (A-B)** Evans Blue dye (EBD) was injected intravenously into wild-type or *Tlr4*^*-/-*^ B6 mice 3 days after intravenous injection of 10 mg/kg DENV2 NS1 (wild-type: blue squares; *Tlr4*^*-/-*^: green triangles; n = 2 per genotype) or 10 mg/kg OVA (wild-type: black circles; n = 2). The dye was allowed to circulate for 3 hours before mice were euthanized. Tissues were harvested, and EBD was extracted in formamide and quantified in **(A)** lungs and **(B)** liver by measuring absorbance at 620 nm against a standard curve.
(TIF)

S6 Fig Related to Fig 4. **DENV2 infection leads to similar levels of morbidity and mortality in *Ifnar^-/-^ and Tlr4^-/-^ x Ifnar^-/-^ mice*. (A-B)** *Ifnar*^*-/-*^ and *Tlr4*^*-/-*^ *x Ifnar*^*-/-*^ B6 mice were injected intravenously with either 10^7^ plaque-forming units (PFU) of DENV2 D220 (straight-lethal, SL; *Ifnar*^*-/-*^: closed green triangles, n = 7; *Tlr4*^*-/-*^ *x Ifnar*^*-/-*^: closed blue triangles, n = 9) or 5 μg of 4G2 (anti-DENV Envelope mAb) 20–24 hours prior to infection with 3 x 10^5^ PFU of D220 (antibody-enhanced, ADE; *Ifnar*^*-/-*^: open green triangles, n = 7; *Tlr4*^*-/-*^ *x Ifnar*^*-/-*^: open blue triangles, n = 9). Mice were then monitored for **(A)** morbidity and **(B)** mortality for 10 days post-infection. **(A)** Mice were observed twice per day and scored for morbidity on a scale of 1 to 5, with 1 being healthy and 5 being moribund. **(B)** Kaplan-Meier survival curve, with data derived from 2 independent experiments. A nonparametric Mantel-Cox log rank test was used to determine significance between groups.
(TIF)

S7 Fig Related to Fig 5. **HMEC-1 express canonical glycocalyx components on the cell surface *in vitro*.** HMEC-1 monolayers were grown for 5 days until confluent on glass cover slips coated with 0.2% gelatin. Monolayers were stained for **(A)** sialic acid (stained with WGA-A647, red), **(B)** chondroitin sulfate (stained with anti-Chondroitin Sulfate mAb CS-56, red), **(C)** heparan sulfate (stained with Heparan Sulfate mAb clone F58-10E4, green), or **(D)** hyaluronic acid (stained with anti-Hyaluronic Acid polyclonal antibody, yellow), and imaged on a Zeiss LSM 710 Axio Observer inverted fluorescence microscope equipped with a 34-channel spectral detector at 20x magnification. **(E)** Merge of **(B-D)**. Images were acquired using the Zen 2010 software (Zeiss). Nuclei were stained with *Hoechst* (blue). Scale bar, 10 μM.
(TIF)
