# Supplementary figures and images for: Dengue virus NS1 cytokine-independent vascular leak is dependent on endothelial glycocalyx components
Source: PLoS Pathog. 2017 Nov 9;13(11):e1006673. doi: 10.1371/journal.ppat.1006673 (PMC5679539; doi:10.1371/journal.ppat.1006673)

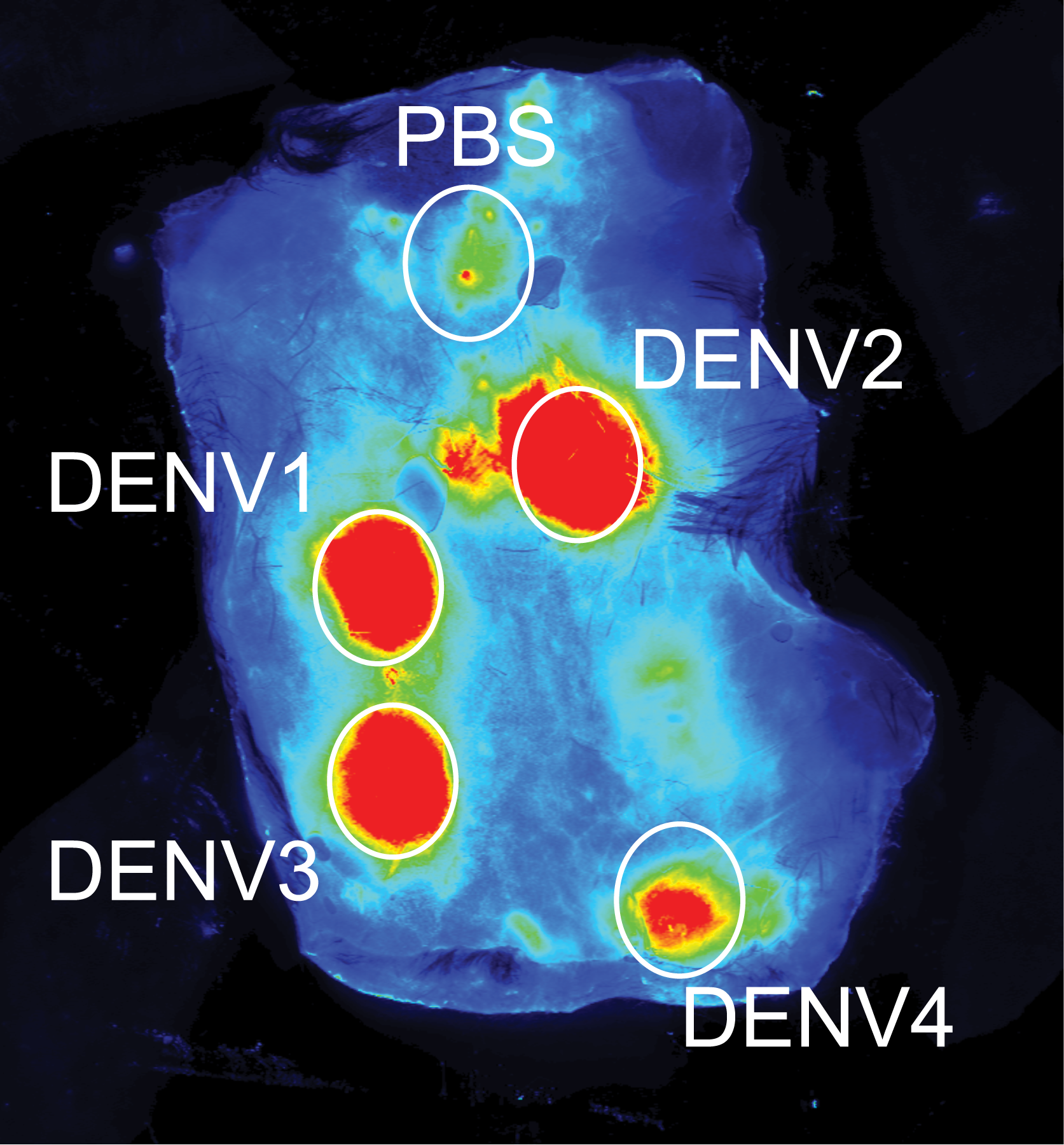

Supplement: S1 Fig — NS1 from DENV1-4 triggers localized vascular leak in the dorsal dermis of mice. Representative image of mouse dorsal dermis following fluorescent dextran assay. Hair was removed from the dorsal dermis of mice, and mice were allowed to recover for 3 days. On the day of the assay, retro-orbital injections of Alexa Fluor 680-conjugated dextran were administered, followed by intradermal injections of PBS, 15 μg DENV1 NS1, 15 μg DENV2 NS1, 15 μg DENV3 NS1, and 15 μg DENV4 NS1. The dermis from each mouse was collected and processed two hours post-injection and scanned using a fluorescent detection system (LI-COR Odyssey CLx Imaging System) at a wavelength of 700 nm, and images were obtained using Image Studio software (LI-COR Biosciences). (TIF) [file ppat.1006673.s001.tif]

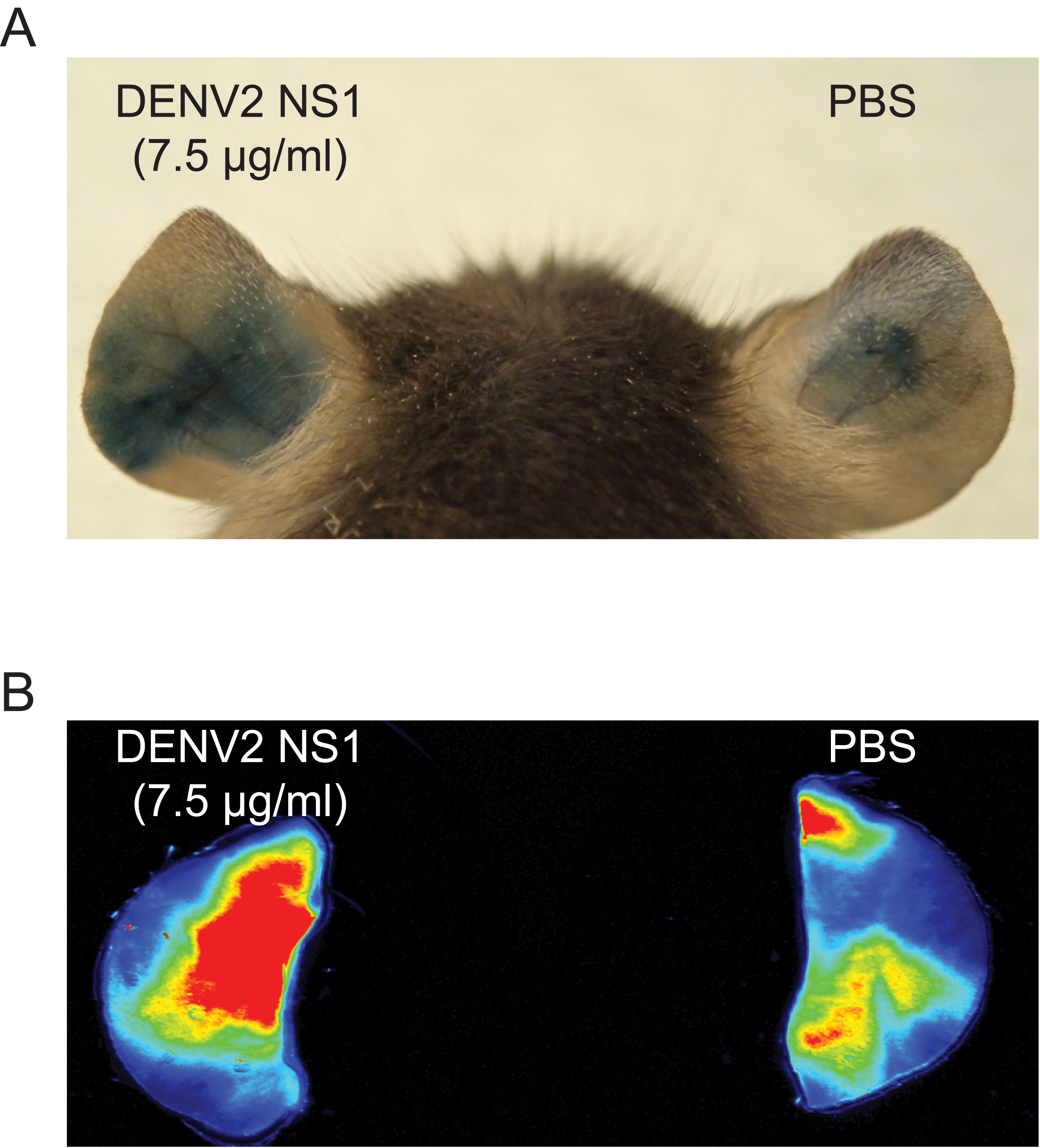

Supplement: S2 Fig — DENV2 NS1 triggers localized vascular leak in the dermis of mouse ears. (A-B) Wild-type B6 mice received intradermal injections with either PBS (right ear) or 7.5 μg DENV2 NS1 (left ear) and, immediately after, intravenous injections of either (A) Evans Blue dye (EBD) or (B) Alexa Fluor 680-conjugated dextran. (A) EBD was allowed to circulate for 30 minutes and ears were photographed. (B) Dextran was allowed to circulate for 2 hours. Ears were removed and scanned using a fluorescent detection system (LI-COR Odyssey CLx Imaging System) at a wavelength of 700 nm, and images obtained using Image Studio software (LI-COR Biosciences). (TIF) [file ppat.1006673.s002.tif]

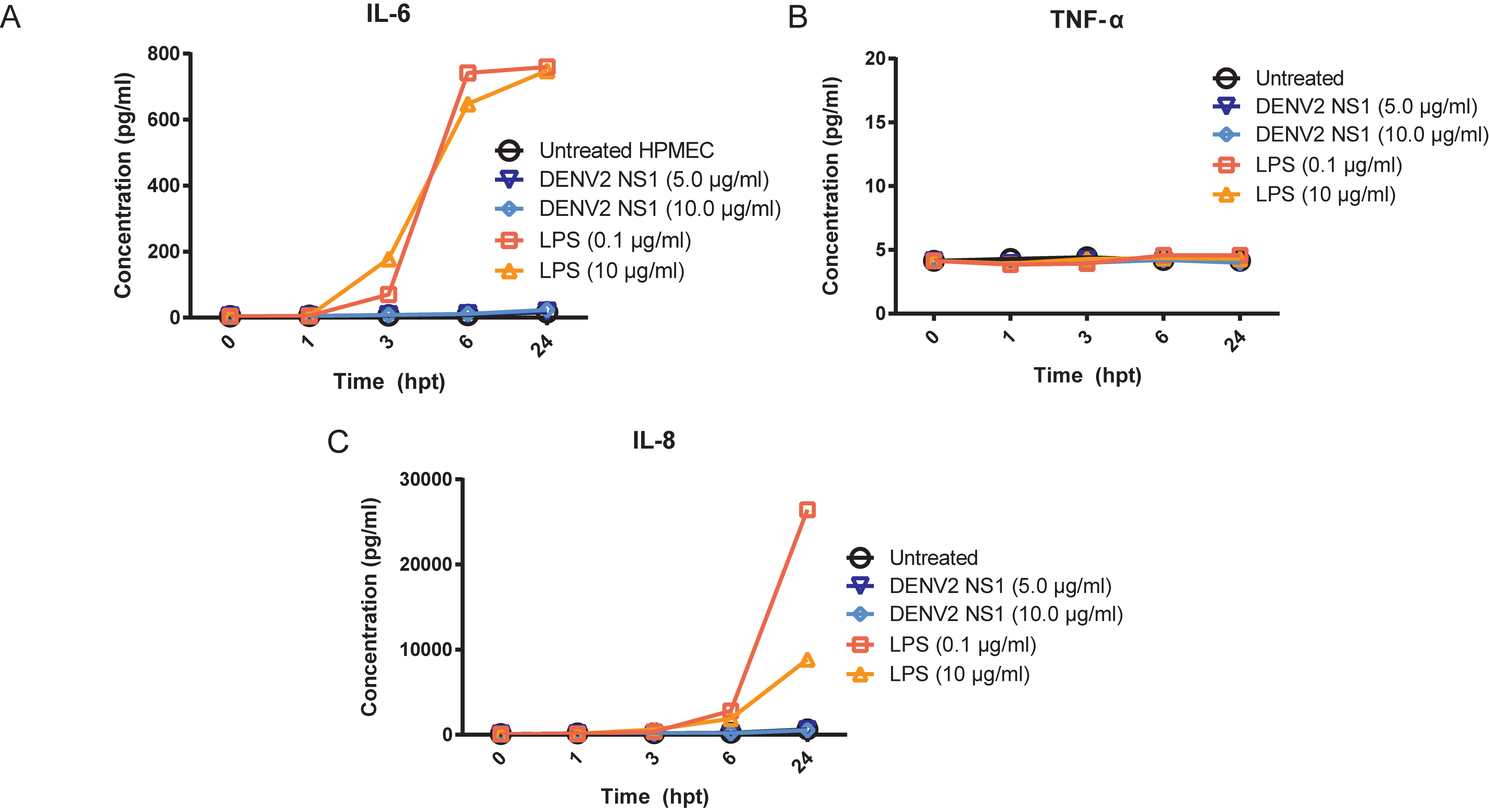

Supplement: S3 Fig — HPMEC do not produce the inflammatory cytokines IL-6, TNF-α, or IL-8 in response to DENV2 NS1 stimulation in vitro. (A-C) HPMEC were stimulated with LPS (0.1 or 10 μg/ml; red squares and orange triangles, respectively) or DENV2 NS1 (5 or 10 μg/ml; dark blue triangles and light blue diamonds, respectively), and supernatant was collected at 0, 1, 3, 6, and 24 hours post-treatment. Untreated HPMEC monolayers were used as a control (black circles). ELISAs for (A) IL-6, (B) TNF-α, and (C) IL-8 were performed on all samples. (TIF) [file ppat.1006673.s003.tif]

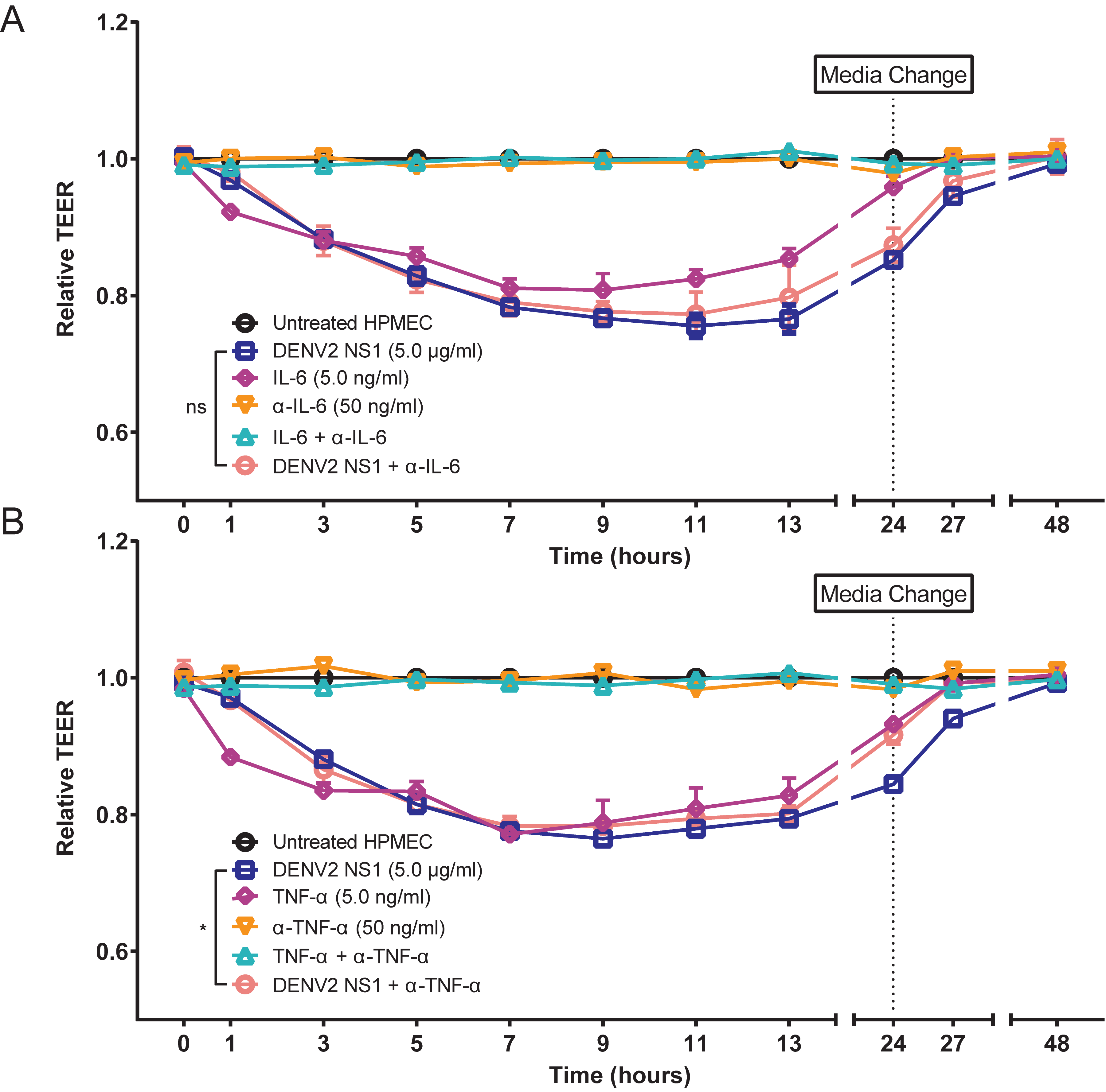

Supplement: S4 Fig — Inflammatory cytokines TNF-α and IL-6 are not involved in DENV2 NS1-induced endothelial hyperpermeability in HPMEC in vitro. (A-B) Trans-endothelial electrical resistance (TEER) of HPMEC monolayers incubated with 5 μg/ml DENV2 NS1 (blue squares), 5 ng/ml recombinant cytokine ((A) IL-6, (B) TNF-α; purple diamonds), 50 ng/ml anti-cytokine mAbs ((A) IL-6, (B) TNF-α; orange triangles), recombinant cytokine + specific mAb ((A) IL-6, (B) TNF-α; green diamonds), or DENV2 NS1 + specific mAb ((A) IL-6, (B) TNF-α; red circles). The background signal was subtracted (using TEER values from a blank Transwell), and data were normalized to untreated HPMEC. All data shown represent the mean +/- SEM and were collected from two independent experiments. Data represent two replicate Transwells per condition. A repeated measure two-way ANOVA was used to determine the significance of anti-cytokine mAbs on DENV2 NS1-induced hyperpermeability in HPMEC. ns = not significant, *P < 0.05. (TIF) [file ppat.1006673.s004.tif]

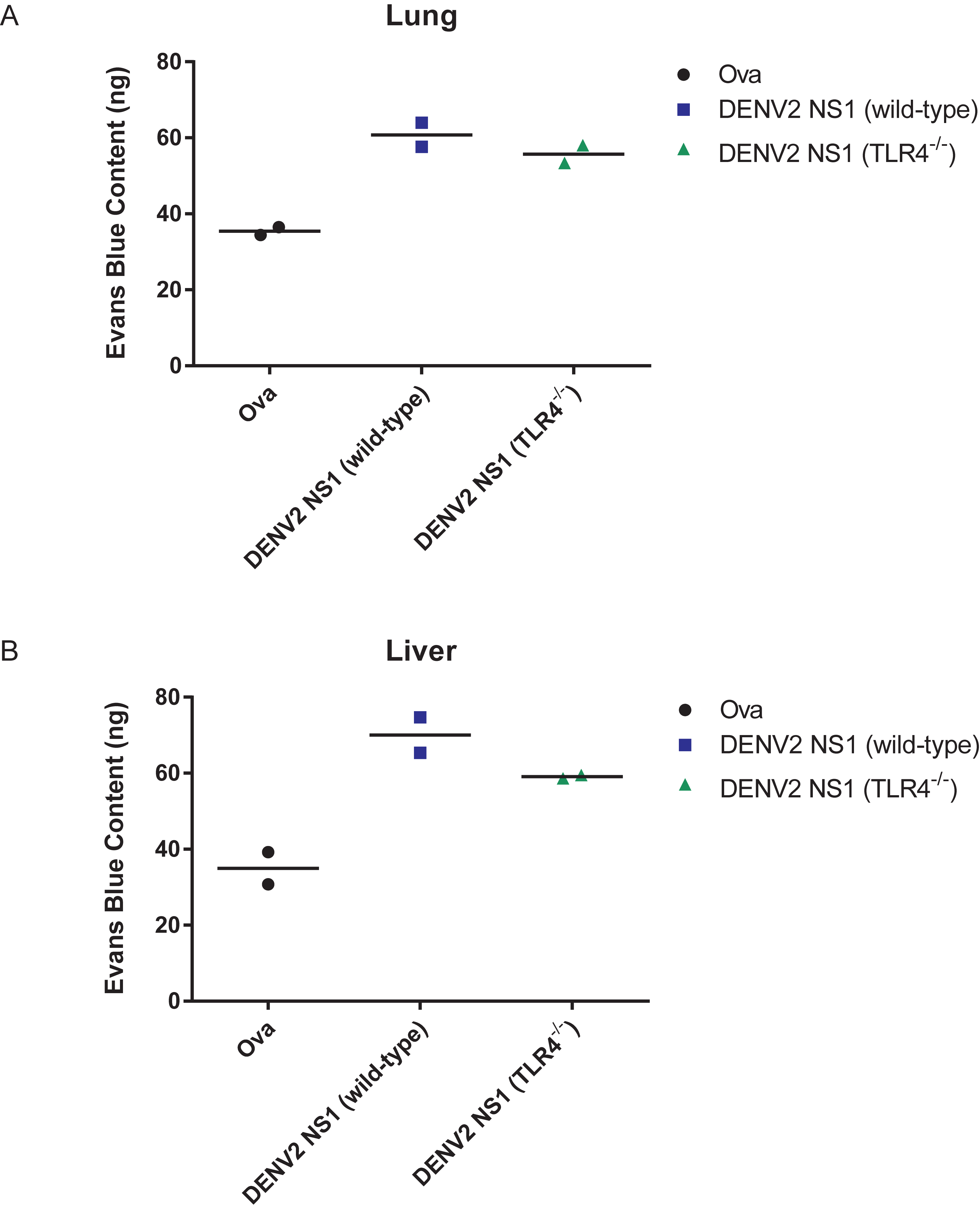

Supplement: S5 Fig — DENV2 NS1-induced systemic vascular leak in vivo is similar in wild-type and TLR4-deficient mice. (A-B) Evans Blue dye (EBD) was injected intravenously into wild-type or Tlr4-/- B6 mice 3 days after intravenous injection of 10 mg/kg DENV2 NS1 (wild-type: blue squares; Tlr4-/-: green triangles; n = 2 per genotype) or 10 mg/kg OVA (wild-type: black circles; n = 2). The dye was allowed to circulate for 3 hours before mice were euthanized. Tissues were harvested, and EBD was extracted in formamide and quantified in (A) lungs and (B) liver by measuring absorbance at 620 nm against a standard curve. (TIF) [file ppat.1006673.s005.tif]

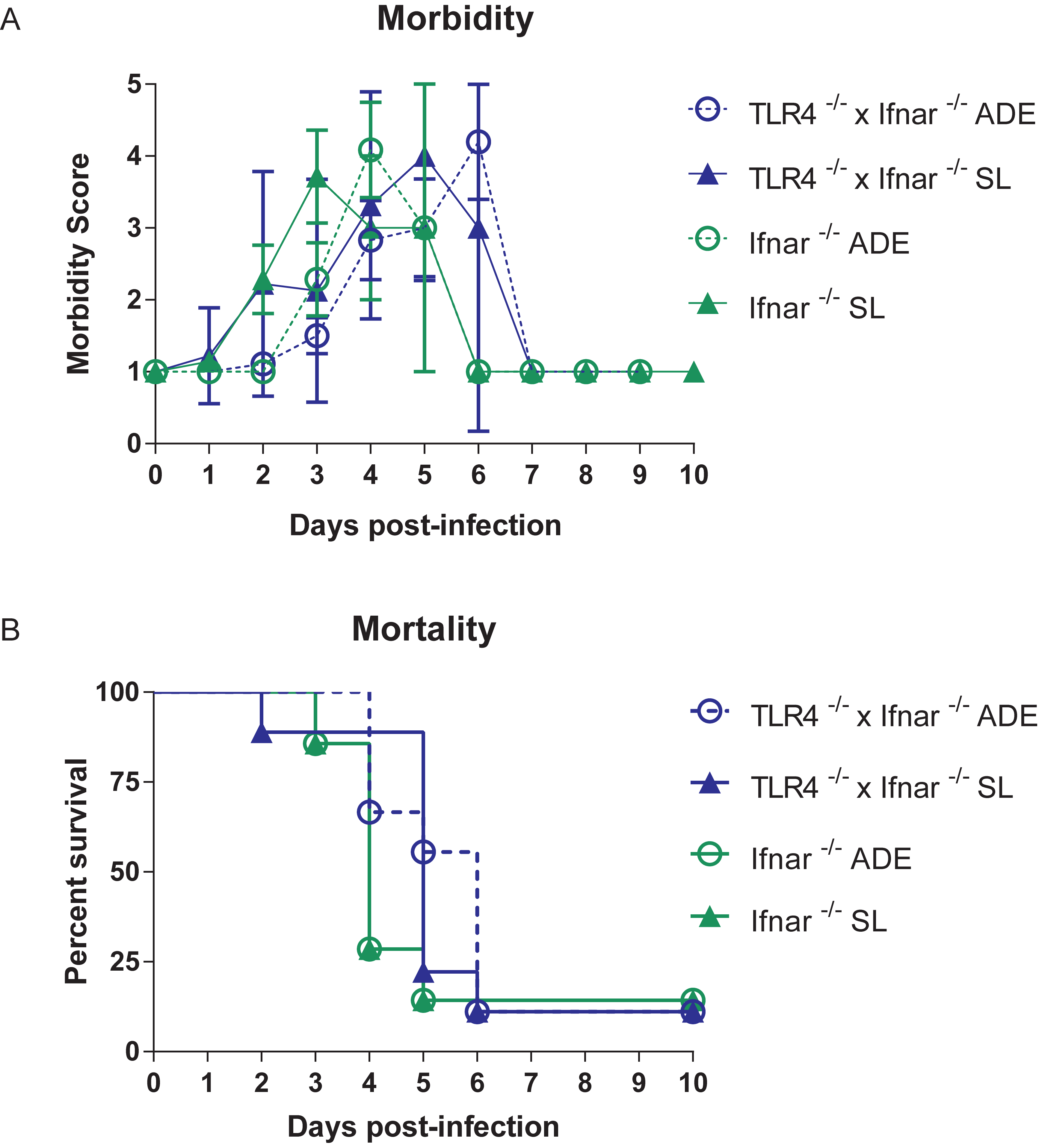

Supplement: S6 Fig — DENV2 infection leads to similar levels of morbidity and mortality in Ifnar-/- and Tlr4-/- x Ifnar-/- mice. (A-B) Ifnar-/- and Tlr4-/- x Ifnar-/- B6 mice were injected intravenously with either 107 plaque-forming units (PFU) of DENV2 D220 (straight-lethal, SL; Ifnar-/-: closed green triangles, n = 7; Tlr4-/- x Ifnar-/-: closed blue triangles, n = 9) or 5 μg of 4G2 (anti-DENV Envelope mAb) 20–24 hours prior to infection with 3 x 10^5^ PFU of D220 (antibody-enhanced, ADE; Ifnar-/-: open green triangles, n = 7; Tlr4-/- x Ifnar-/-: open blue triangles, n = 9). Mice were then monitored for (A) morbidity and (B) mortality for 10 days post-infection. (A) Mice were observed twice per day and scored for morbidity on a scale of 1 to 5, with 1 being healthy and 5 being moribund. (B) Kaplan-Meier survival curve, with data derived from 2 independent experiments. A nonparametric Mantel-Cox log rank test was used to determine significance between groups. (TIF) [file ppat.1006673.s006.tif]

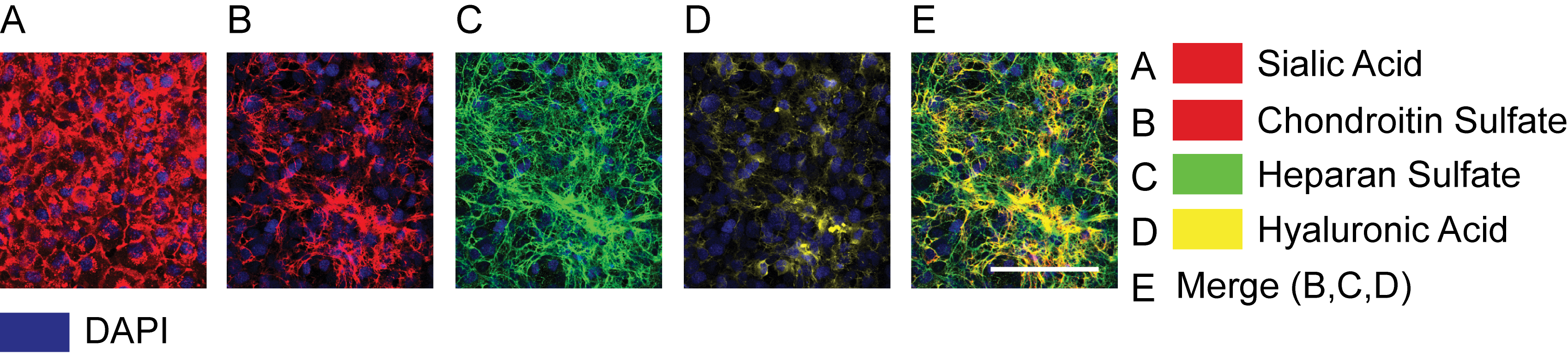

Supplement: S7 Fig — HMEC-1 express canonical glycocalyx components on the cell surface in vitro. HMEC-1 monolayers were grown for 5 days until confluent on glass cover slips coated with 0.2% gelatin. Monolayers were stained for (A) sialic acid (stained with WGA-A647, red), (B) chondroitin sulfate (stained with anti-Chondroitin Sulfate mAb CS-56, red), (C) heparan sulfate (stained with Heparan Sulfate mAb clone F58-10E4, green), or (D) hyaluronic acid (stained with anti-Hyaluronic Acid polyclonal antibody, yellow), and imaged on a Zeiss LSM 710 Axio Observer inverted fluorescence microscope equipped with a 34-channel spectral detector at 20x magnification. (E) Merge of (B-D). Images were acquired using the Zen 2010 software (Zeiss). Nuclei were stained with Hoechst (blue). Scale bar, 10 μM. (TIF) [file ppat.1006673.s007.tif]
